# Novel indazolylchromones: synthesis, fungicidal evaluation, molecular docking and aquatic toxicity prediction

**DOI:** 10.3389/fchem.2024.1411187

**Published:** 2024-06-11

**Authors:** Riya Kundu, Najam Akhtar Shakil, Neethu Narayanan, Deeba Kamil, Virendra Singh Rana, Kailash P. Tripathi, Parshant Kaushik

**Affiliations:** ^1^ Division of Agricultural Chemicals, ICAR-IARI, New Delhi, India; ^2^ The Graduate School, ICAR-IARI, New Delhi, India; ^3^ Division of Plant Pathology, ICAR-IARI, New Delhi, India

**Keywords:** indazolylchromones, NMR, fungus, molecular docking, ECOSAR

## Abstract

Fungal diseases cause substantial loss to agricultural crops, affecting both quantities and quality. Although several methods are used for preventing disease incidence, fungicides remain crucial for higher yields and better quality. But in the past, the efficacy of several fungicides has decreased due to increased cases of fungicide resistant. In our pursuit of new effective fungicides, we synthesised a series of twenty 2-Indazol-1-yl-chromen-4-one derivatives (6a- 6t). The characterization of synthesized compounds was performed by several spectroscopic methods including Infrared, Nuclear Magnetic Resonance (^1^H and ^13^C) and HRMS. Out of 20 synthesised compounds, 19 (6b- 6t) were found to be novel. All synthesised indazolylchromones showed very good antifungal activity against *Sclerotium rolfsii* and *Fusarium oxysporum*. Among the tested compounds, **6t** and **6f** exhibited very good fungicidal activity against *S. rolfsii* with an ED_50_ of 10.10 mg L^-1^ and 16.18 mg L^-1^, respectively. In case of *Fusarium oxysporum* compound **6f** displayed good’ activity with an ED_50_ value of 27.82 mg L^-1^. Molecular docking study was done to predict the binding sites of most active compounds, **6t** and **6f** with Cytochrome P450 14alpha -sterol demethylase (CYP51) enzyme using molsoft software. The acute toxicity predictions the of synthesized compounds for fish (LC_50_,96 Hr), daphnid (LC_50_, 48 Hr) and green algae (EC_50_, 96Hr) and the chronic toxicity predictions (ChV) were assessed using Ecological Structure Activity Relationship (ECOSAR) model. As per ECOSAR prediction, all the chemicals are inside AD and not missing predictions.

## 1 Introduction

Fungi represent one of the most harmful groups of phytopathogens, responsible for 80% of plant diseases (Agrios, 2005). *Sclerotium rolfsii* is a soil-borne fungus that generally occurs worldwide in hot and moderately warm places causing disease on hundreds of plant species. Typically, *S. rolfsii* attacks the plant near the surface of soil (Kator et al., 2015). The common diseases caused by *S. rolfsii* are foot rot, stem rot, root rot and wilt. Another important soil borne fungal pathogen is *Fusarium oxysporum* which causes fusarium wilt, a serious threat for agriculture (Fisher et al., 2012). *F oxysporum* is among the top ten most destructive fungal plant pathogens in the world (Dean et al., 2012). *F oxysporum* produces chlamydospores in the soil and survive for a longer time. It penetrates the roots, spread throughout the tissues, interfere with xylem and hinders waterflow resulting in wilting of plants.

Fungicides have been used for over two centuries for managing plant diseases, but in the recent past, many instances of fungicide resistance have led to the loss of many important fungicides (Hollomon, 2015). As a result, synthesis of new molecules has become increasingly important for crop protection. One of the approaches to address the problem of fungicide resistance is the development of hybrid fungicide molecules by combining different scaffolds. The hybrid molecules have multiple sites of action which minimizes the chances of fungicide resistance.

Chromones are natural compounds, mainly obtained from plants. Chromone nucleus is a widely distributed pharmacophore in various important synthetic and natural drugs (Simin et al., 2002; Yadav et al., 2005; Aggarwal et al., 2013; Stompor et al., 2015; Badawy et al., 2015; Kaushik et al., 2021).

Azole fungicides have been widely used in agriculture are considered to be moderate risk fungicides for resistance development. Indazole is one of the important class of azoles. Indazoles are very rare in nature but a large number of synthetic indazole derivatives has been reported to have herbicidal activity (Hwang et al., 2005), insecticidal activity (Lahm et al., 2002), and fungicidal activity (Shakil et al., 2013; Tang et al., 2019). Thus, in the present study we synthesized indazolylchromones by combining the indazole moiety with chromones.

## 2 Experimental

### 2.1 Chemicals and instruments

The chemicals required for synthesis of indazolylchromones were obtained from manufacturers. All the chemicals were utilized as received and not purified unless stated otherwise. The TLC plates (20 × 20 cm) coated with silica gel (having F254 fluorescence indicator) were used for reaction monitoring. 100–200 mesh size silica gel was used for column chromatography. Melting points of synthesised compounds were measured by Buchi M-560, reported values were not adjusted/corrected. NMR spectra of (^1^H and ^13^C) of synthesised compounds were recorded by JEOL JNM-ECZ400/SI. The mass spectrometry was carried out by ABSCIEX triple TOFTM 5600+ having Turbo Ion Spray. ED_50_ values were estimated with the SPSS statistical package. Molecular docking was carried out using molsoft software.

### 2.2 Synthesis of 2-indazol-1-yl-chromen-4-one derivatives (6a- 6t)

**SCHEME 1:**
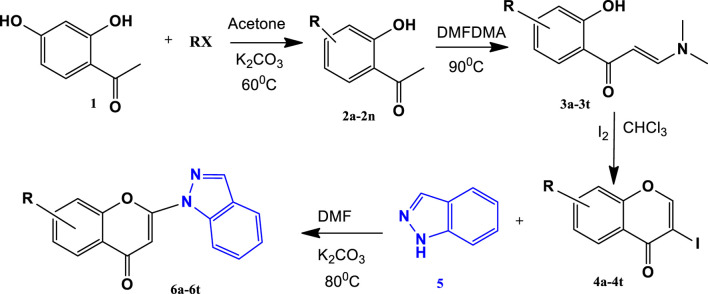
Synthesis of 2-Indazolyl-chromone (Gamill, 1979; Sugita et al., 1996; Kaushik et al., 2021).

2-hydroxyacetophenone derivatives **2a-2n** were synthesised by reacting 2- hydroxyacetophenone **1** and bromoalkanes or iodoalkanes of different chain lengths in a molar ratio of 1:1.2 by continuous stirring for 6 h at 60 °C in the presence of K_2_CO_3_ and acetone. These 2-hydroxyacetophenone derivatives **2a-2t** (1 mmol) on reaction with *N,N*-dimethylformamidedimethylacetal (DMFDMA) (2 mmol, 2 molequiv) overnight at 90 °C resulted in formation of enaminones **3a-3t**. The chromones **4a-4t** were synthesized by reacting enaminones **3a-3t**, (1 mmol) and iodine (2 mmol, 2 molequiv), in 30 mL CHCl_3_ at room temperature for 8 h with continuous stirring. The pure chromones were obtained in 67%–89% yield by column chromatography of synthesised crude compounds. Finally, conjugated addition of 3-iodochromones **4a-4t** with indazole **5** gave 2-Indazolyl-chromones **6a-6t** in the yield range of 76%–94%. (Scheme 1, Table 1). All synthesized 2-Indazol-1-yl-chromen-4-one derivatives were characterized using IR, ^1^H-NMR, and ^13^C-NMR analysis. The spectral analysis of all synthesised compounds has been provided in supporting document.

**TABLE 1:** Structures of the synthesized indazolylchromones (6a-6t).

Sr.No.	Structure	Yield %
1	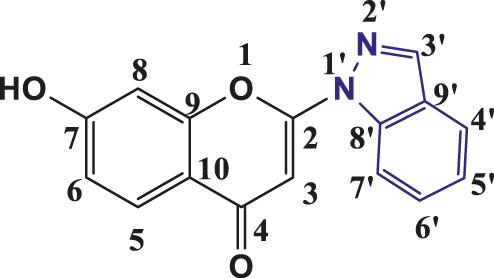 **6a**	86
2	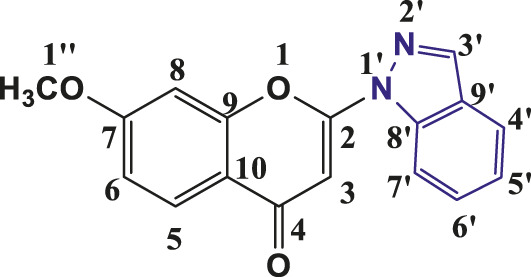 **6b**	89
3	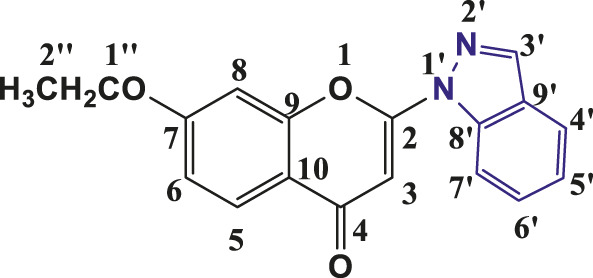 **6c**	86
4	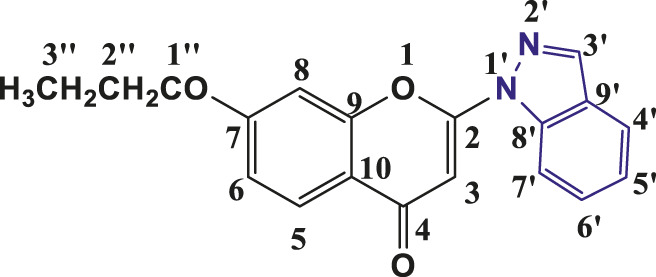 **6d**	80
5	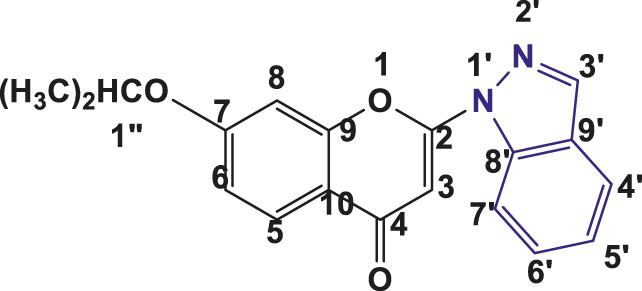 **6e**	88
6	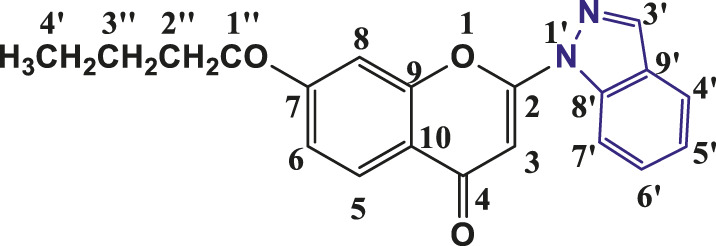 **6f**	94
7	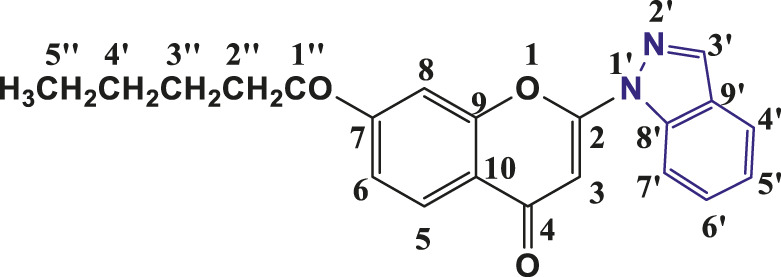 **6g**	83
8	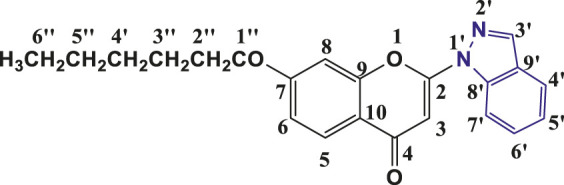 **6h**	92
9	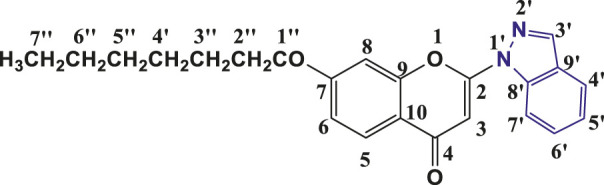 **6i**	88
10	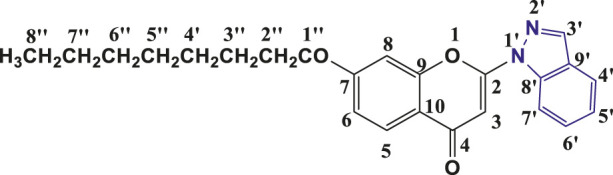 **6j**	89
11	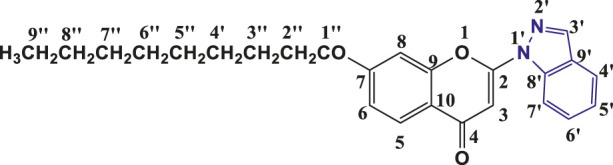 **6k**	82
12	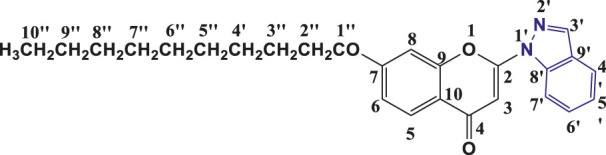 **6l**	76
13	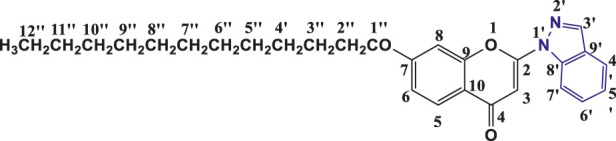 **6m**	80
14	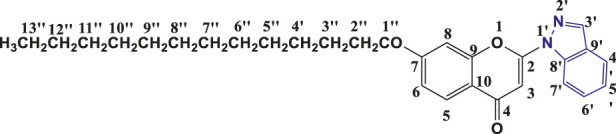 **6n**	82
15	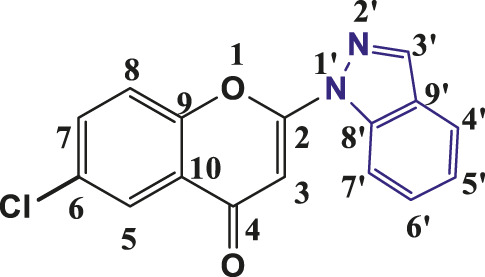 **6o**	77
16	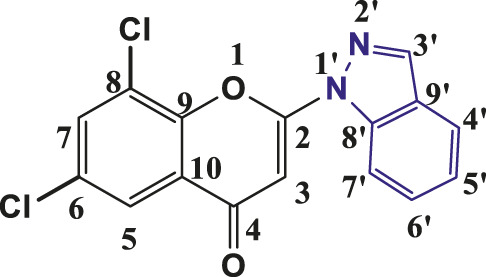 **6p**	81
17	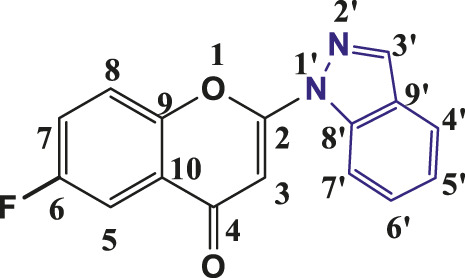 **6q**	79
18	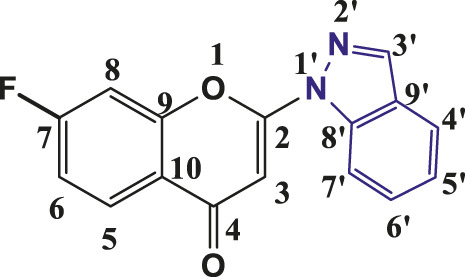 **6r**	82
19	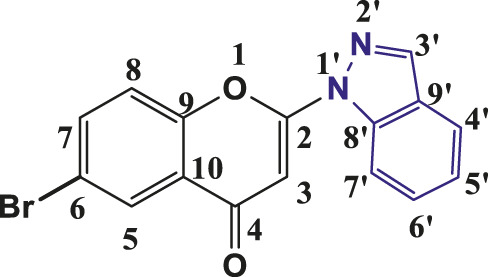 **6s**	84
20	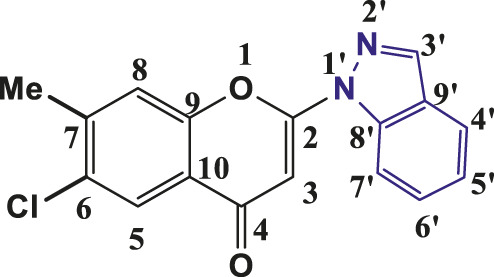 **6t**	84

### 2.3 Bioefficacy evaluation

#### 2.3.1 Test fungus

Two fungal strains, *F. oxysporum* ITCC8113 and *S. rolfsii* ITCC 6866, were obtained from the Indian Type Culture Collection (ITCC) centre, Division of Plant Pathology, ICAR-Indian Agricultural Research Institute, New Delhi, India. The strains were kept at a temperature of 27 °C for 4–7 days on Potato Dextrose Agar (PDA) slants. Before conducting the fungicidal bioassay, sub-culturing was performed on Petri plates.

#### 2.3.2 *In vitro* fungicidal activity

A stock solution of each compound, with a concentration of 10,000 mg L^-1^, was prepared using DMSO (Dimethylsulphoxide). From the stock solution, five different test concentrations were obtained through serial dilution, namely, 100 mg L^-1^, 50 mg L^-1^, 25 mg L^-1^, 12.5 mg L^-1^, and 6.25 mg L^-1^ for *S. rolfsii* and at 200 mg L^-1^, 100 mg L^-1^, 50 mg L^-1^, 25 mg L^-1^ and 12.5 mg L^-1^ for *F. oxysporum.* Commerical available fungicide Hexaconazole 5% SC and Carbendazim 50% WP were used as positive control.

The fungicidal bioassay was conducted in a laboratory setting using the poisoned food technique (Nene et al., 1979). To determine the effectiveness of the antifungal treatment, the percentage of growth inhibition was computed using Abbott’s formula (Abbott, 1925).

To calculate the corrected % inhibition (IC), the following formula was employed:

$$\text{IC}=\left(I-\text{CF}\right)/\left(100-\text{CF}\right)\;x\;100$$

I denote the % inhibition, CF = (90 - C)/C x 100, where 90 is the Petriplate diameter (in mm) and C represents the growth of mycelium (in mm) in control.

The ED_50_ (mg L^-1^) values, which indicate the Effective Dose for 50% inhibition, were determined using the SPSS statistical package (v16.0).

### 2.4 Molecular docking

Molecular docking was carried out to predict the interaction of most active compounds, **6t** and **6f** with Cytochrome P450 14alpha -sterol demethylase (CYP51) enzyme using molsoft software and the respective docking scores were calculated to compare the interaction of compounds with the lanosterol 14α-demethylase (LDM) enzyme.

### 2.5 Aquatic toxicity prediction

The aquatic toxicity potentials of the synthesized compounds were assessed using Ecological Structure Activity Relationship (ECOSAR) model. It is a free and easy to use computer programme developed by US EPA. It predicts the median lethal concentration (LC_50_), median effective concentration (EC_50_), and chronic value (ChV) for various species.

## 3 Result and discussion

### 3.1 Synthesis and characterization of individual compounds

In the present work, total twenty indazolylchromones (6a- 6t) were synthesized, out of which 19 compounds (6b- 6t) were found novel. The indazolylchromones were synthesized by 4 steps synthetic scheme (scheme- I). In the last step, conjugated addition of 3-iodochromones **4a-4t** with Indazole **5** is the Michael type addition reaction where indazole act as Michael donor (nucleophile) and chromone as Michael acceptor. In the first step indazole attacks chromone ring at 2-position and gives an intermediate **A,** which on removal of H-1 results in the formation of 2-(1-Indazolyl) chromones (Sugita et al., 1996).

The above synthetic method resulted in the synthesis of 2-(1-Indazolyl) chromones in 76%–94% yield. All the synthesised compounds were characterised by IR, ^1^H NMR and ^13^C NMR. In all synthesised compounds **6a-6t,** typical peak at δ 6.83–6.93 (1H, s, H-3) appeared due to elimination of iodine from 3- Iodochromones. The peaks at δ 8.21–8.35 (2H, s, H- 3′), δ 7.74–8.19 (1H, d, J = 8, H-4′), δ 7.31–7.48 (1H, t, J = 8, H-5′), δ 7.55–7.67 (1H, t, J = 7.2, H- 6′) and δ 7.66–7.85 (1H, d, J = 8, H-7′) were assigned to protons of indazolyl ring in ^1^H-NMR spectra of all the compounds and validates the synthesis of indazolylchromones. In ^13^C-NMR, the peaks at δ 96.10–96.42 (C-3), 123.76–125–85 (C-5′), 121.76–123.99 (C-4′), 155.20–157.96 (C-2), 129.06–135.57 (C- 6′), 114.31–119.07 (C-7’) and at δ 176.44–177.23 for carbonyl carbon (C=O) were prominent for all the synthesised compounds. In IR spectra stretching of 1,675–1,677 (C=O), 1,365–1,368 (C-N) and 1,532–1,537 (pyrone ring C=C stretch) supported the NMR data. The spectroscopic data revealed that in the above-synthesized compounds, the N atom at the one-position of the indazole was linked to chromone ring at C-2 position.

### 3.2 *In vitro* antifungal activity

**FIGURE 1:**
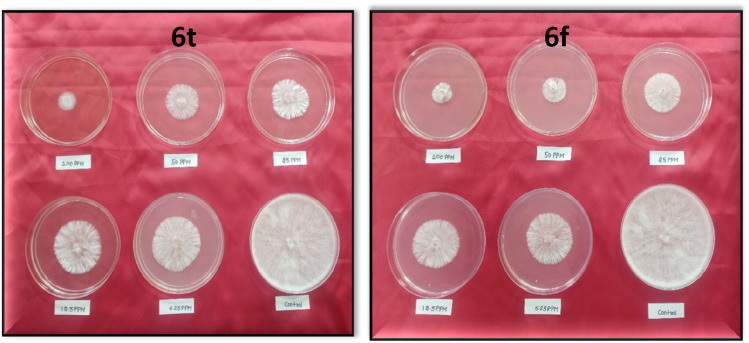
*In vitro* antifungal activity of 6t and 6f against *Sclerotium rolfsii*.

**FIGURE 2:**
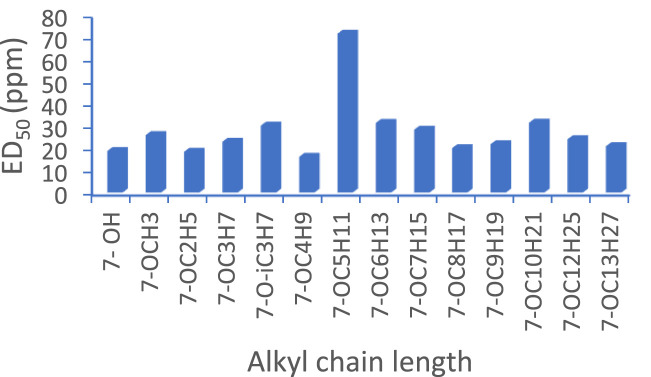
*In vitro* antifungal activity of alkoxy derivatives.

All the synthesised indazolylchromones (6a- 6t) exhibited antifungal activity against *S. rolfsii* (Table 2), but compound 6t (Figure 1) exhibited the highest activity (ED_50_ = 10.10 mg L^-1^) and performed at par with that of Hexaconazole 5% SC (ED_50_ = 8.57 mg L^-1^), a commercial fungicide. Among the alkoxy derivatives of indazolylchromones (6a- 6n), butoxy derivative 6f, 2-Indazol-1-yl-7-butoxy chromen-4-one, was found most active with ED_50_ = 16.18 mg L^-1^. It was observed that alkoxy derivatives having even number of carbon chain length were more active as compared to odd number of carbon chain length. This trend was observed up to carbon chain length of C- 6. After C-6 there was no regular trend was observed (Figure 2).

**TABLE 2:** *In vitro* antifungal activity of synthesized compounds against *S. rolfsii*.

Compounds	ED_50_ (mgL^-1^)	χ2	Regression equation	Fiducial Limit
6a	18.82	2.807	1.12x + −1.24	13.57–24.94
6b	26.06	3.468	0.8x + −1	16.33–42.54
6c	18.48	4.53	0.96x + −1.12	11.83–26.46
6d	23.03	4.10	0.96x + −1.12	15.07–34.32
6e	30.35	0.825	0.96x + −1.32	21.11–46.23
6f	16.18	8.614	1.6x + −1.7	4.50–31.87
6g	71.72	4.22	1.66x + −3	30.69–176.67
6h	31.61	1.09	1.28x + −1.885	24.75–38.92
6i	28.52	0.334	0.8 x + −1.2	20.06–42.00
6j	20.10	0.812	1.12 x + −1.44	15.26–25.77
6k	21.94	0.08	1.12 x + −1.44	16.37–28.84
6l	31.68	0.385	1.6x + −2.2	25.36–40.49
6m	24.17	1.60	1x + −1.25	18.05–32.22
6n	21.03	0.874	1.12x + −1.44	15.16–28.26
6o	30.25	0.66	1.12x + −1.64	23.83–39.22
6p	29.55	4.19	0.8x + −1	17.44–56.19
6q	19.52	1.640	0.96 + −1.12	13.60–26.65
6r	48.36	0.923	0.71 x + −1.11	32.69–70.95
6s	28.48	2.16	0.8x + −1	18.65–45.91
6t	10.105	2.87	1.12x + −1.04	6.30–13.87
Hexaconazole 5% SC	8.57	0.325	0.75 x + −0.78	6.08–10.22

**FIGURE 3:**
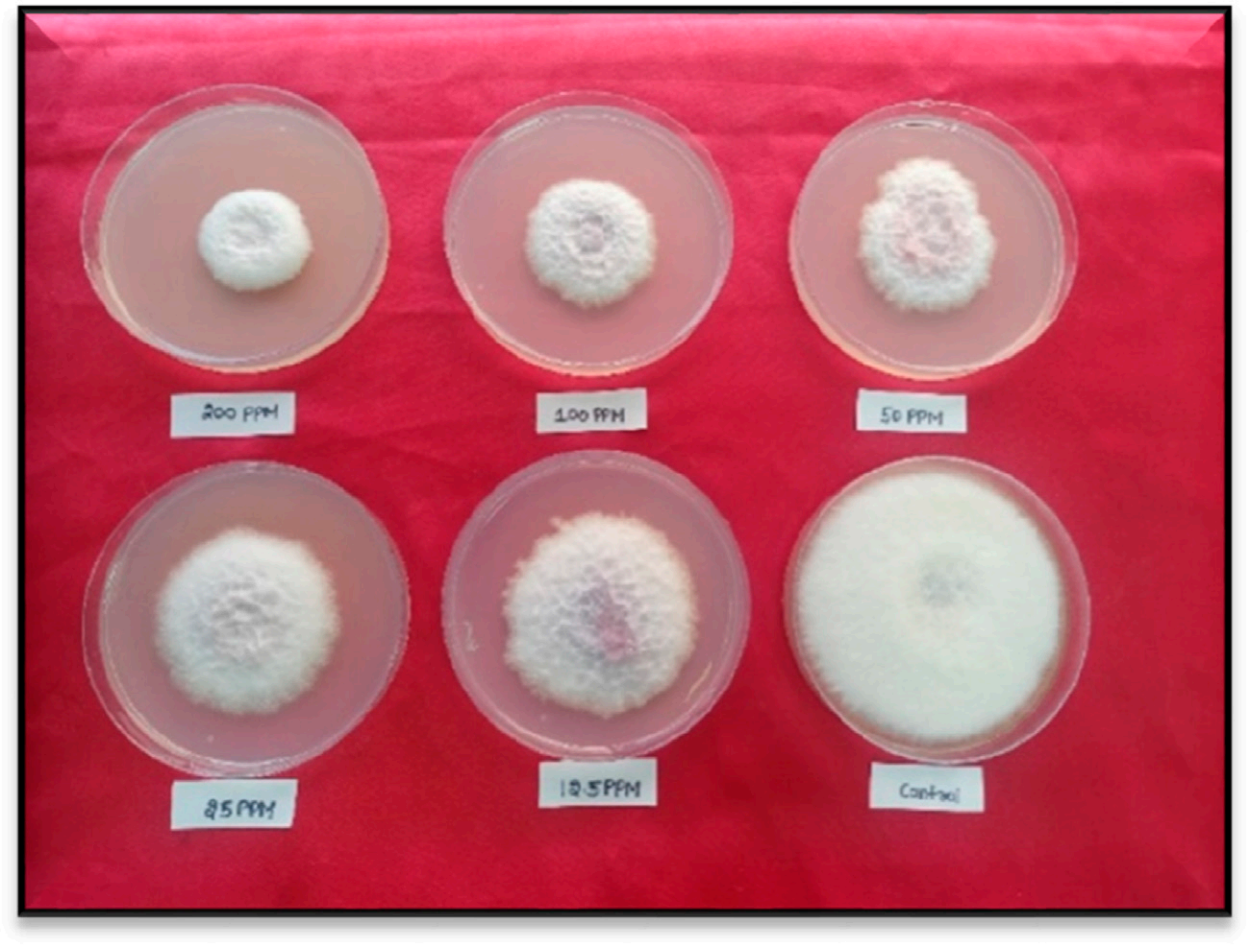
*In vitro* antifungal activity of 6f against *Fusarium oxysporum*.

In case of *F*. *oxysporum*, all the indazolylchromones (6a- 6t) showed antifungal activity (Table 3), compound 6f with ED_50_ = 27.82 mg L^-1^was found most active (Figure 3), but that was less than commercial fungicide carbendazim 50% WP (ED_50_ = 9.01 mg L^-1^). Antifungal activity of synthesised compounds (6a- 6t) against *F. oxysporum* was less than their antifungal activity against *S. rolfsii.*

**TABLE 3:** *In vitro* antifungal activity of synthesized compounds against *F. oxysporum*.

Compounds	ED_50_ (mgL^-1^)	χ2	Regression Equation	Fiducial Limit
6a	71.32	0.36	0.7143 x + −1.3143	46.74–127.37
6b	75.99	1.745	0.8571 x + −1.6571	55.82–112.67
6c	56.27	0.80	1 x + −1.8	42.28–76.42
6d	76.64	0.957	0.8929 x + −1.6429	56.43–113.52
6e	95.08	1.06	1.0714 x + −2.0714	73.08–134.40
6f	27.82	6.71	0.96 x + −1.12	7.49–182.29
6g	50.82	0.541	0.8571 x + −1.4571	37.37–69.31
6h	54.86	0.885	0.8929 x + −1.6429	41.73–73.06
6i	70.05	0.171	0.5714 x + −1.0714	44.73–130.01
6j	51.07	0.117	0.5714 x + −0.9714	33.07–79.40
6k	120.98	0.164	0.7143 x + −1.5143	79.30–256.90
6l	89.29	2.35	1 x + −2	69.72–122.21
6m	70.37	0.74	1 x + −1.8	54.29–95.64
6n	86.20	0.702	0.8571 x + −1.6571	63.22–131.21
6o	159.69	1.226	0.8571 x + −1.8571	102.99–305.85
6p	59.03	034	0.8929 x + −1.6429	43.43–82.85
6q	41.85	1.01	0.8571 x + −1.4571	29.20–57.68
6r	35.35	0.61	0.7143 x + −1.1143	21.29–51.97
6s	99.38	1.78	0.7143 x + −1.5143	67.64–182.33
6t	96.12	0.833	0.56 x + −1.02	49.21–678.32
Carbendazim 50% WP	9.01	1.56	0.95 x + - 1.15	3.21–22.87

### 3.3 Molecular docking

The docking study of most potent compounds, 6f and 6t revealed that both compounds have different orientations, which in turn results in different modes of binding in the active site of CYP51.

**FIGURE 4:**
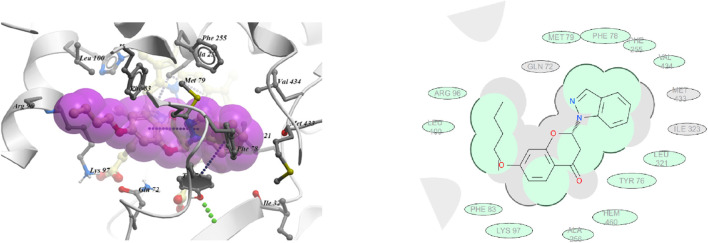
3D and 2D images of compound 6f with target protein CYP51.

In Figure 4, it has been observed that in compound **6f**, the indazole ring is having π-π with TRY 76 and hydrophobic interactions with MET 79, PHE 255, PHE 78, VAL 434, LEU 321 residues. The chromone ring was found to have hydrophobic interactions with ALA 256 and HEM 460 residues. The butoxy chain of chromone ring was observed to be engaged in hydrophobic interactions with LYS 97, PHE 83, LEU 100 and ARG 96 residues.

**FIGURE 5:**
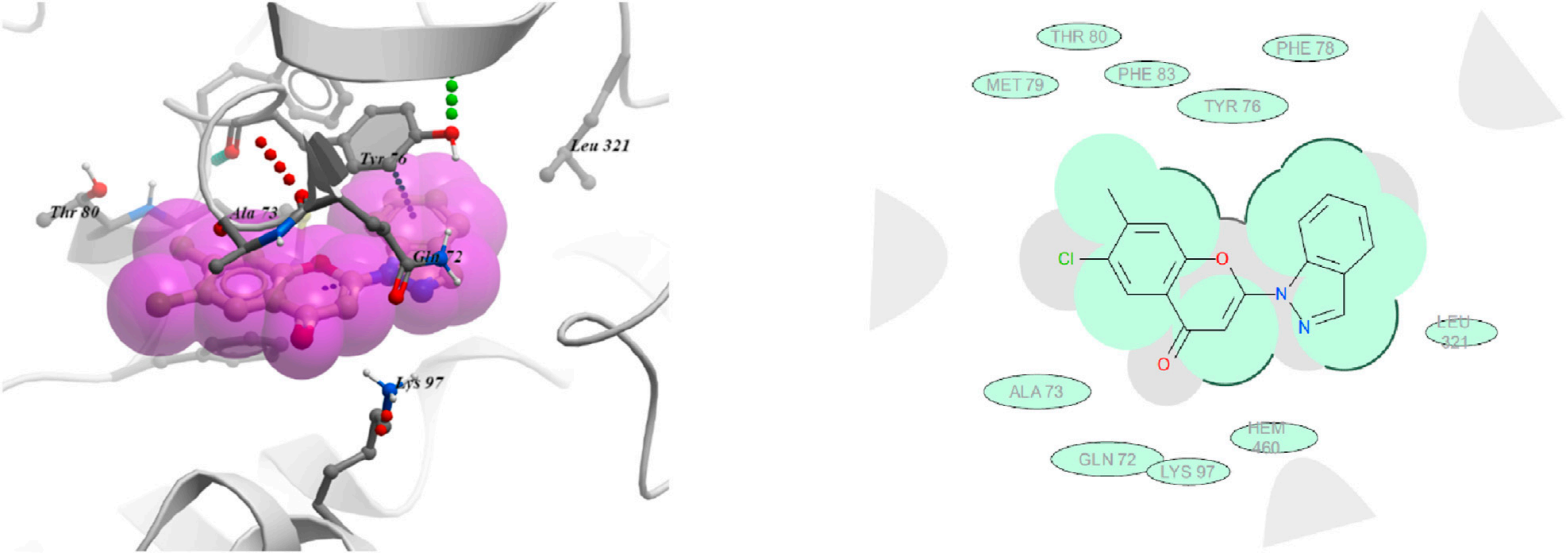
3D and 2D images of compound 6t with target protein CYP51.

In case of compound **6t** (Figure 5), it has been observed that the indazole ring is having π-π with TRY 76 and hydrophobic interactions with PHE 78, LEU 321and HEM 460 residues. The chromone ring was found to have hydrophobic interactions with GLN 72, ALA 73, MET 79 THR 80 and PHE 83 residues.

**FIGURE 6:**
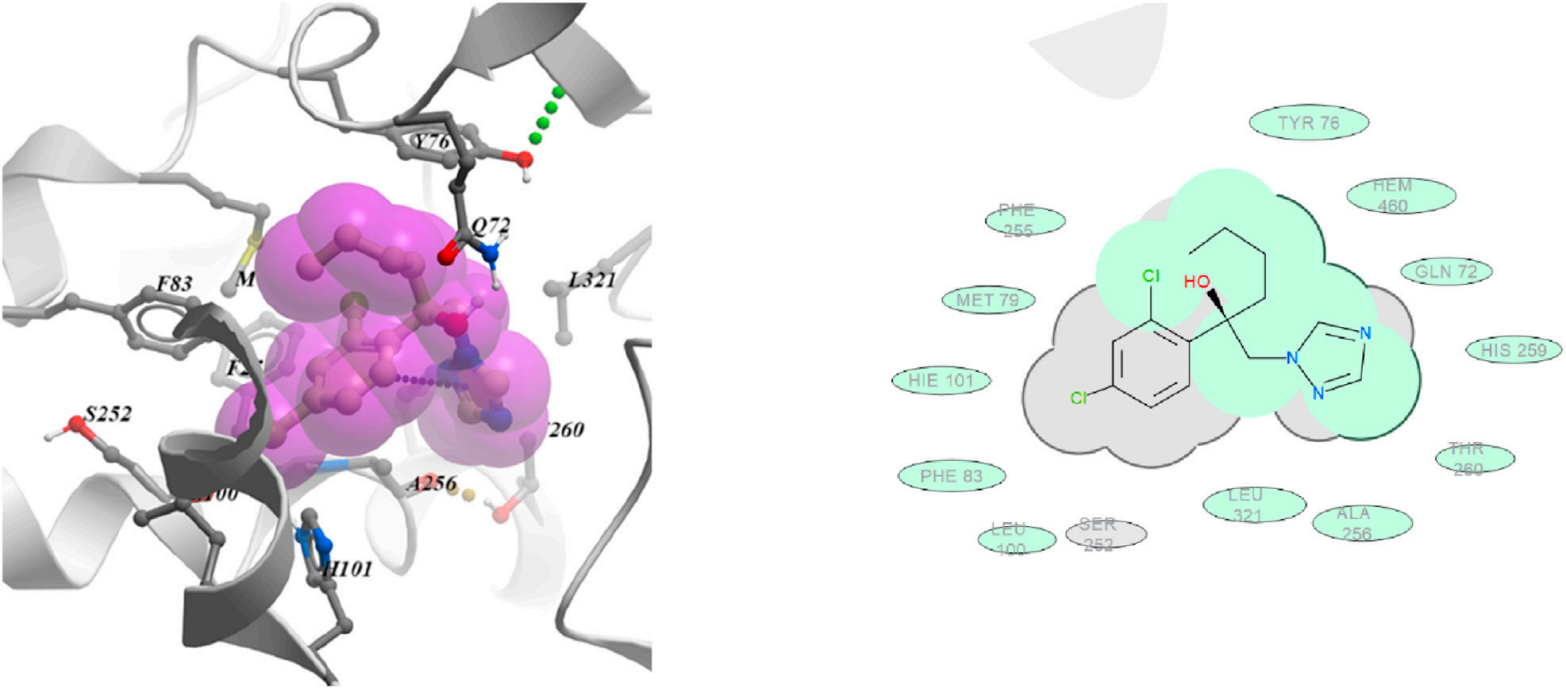
3D and 2D images of hexaconazole with target protein CYP51.

In commercial fungicide hexaconazole (Figure 6), triazole ring showed hydrophobic interaction with HEM 460, HIS 259, THR 260 and ALA 256 residues. The benzyl ring was found to be engaged with PHE 255, HIE 101 PHE 83 and LEU 100 residues.

The docking studies revealed that compound **6t** with highest negative binding energy value (−17.77 kcal mol^−1^) showed better binding with lanosterol enzyme as compared to compound **6f** (−17.65 kcal mol^−1^). These docking studies supports the *in vitro* bioassay result where compound **6t** (ED _50_ = 10.10 mg L^-1^) exhibited higher fungicidal activity than compound **6f** (ED _50_ = 16.18 mg L^-1^).

### 3.4 Aquatic toxicity

**FIGURE 7:**
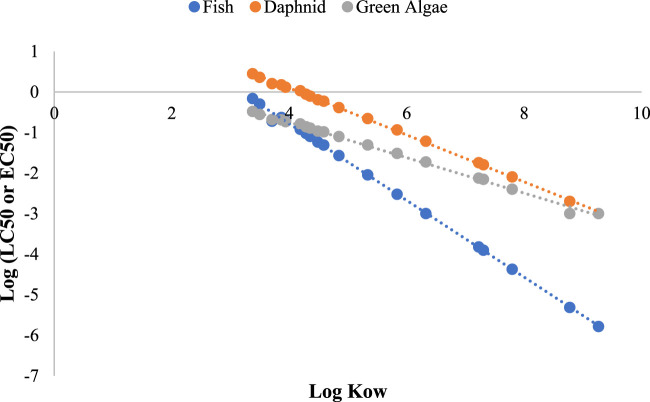
Plot of log LC_50_ (fish and daphnia) or EC_50_ (green algae) values against log K_ow_.

**FIGURE 8:**
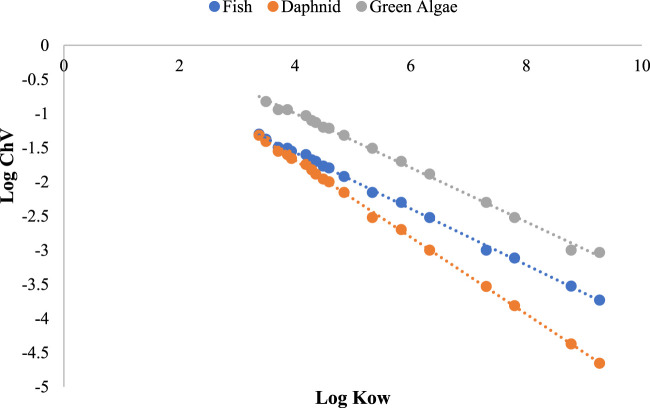
Plot of log ChV values against log K_ow_.

The acute toxicity predictions for fish (LC_50_,96 Hr), daphnid (LC_50_, 48 Hr) and green algae (EC_50_, 96Hr) and the chronic toxicity predictions (ChV) were compared and tabulated in table 5. Fish LC_50_ values varied from 1.64 × 10^−6^ to 0.685 mg/L whereas the chronic toxicity values varied from 1.86 × 10^−4^ to 0.042 mg/L among the different compounds, the highest toxicity was shown by the alkoxy substituted compounds. Among the alkoxy substituted compounds, the toxicity was found to be increasing with increase in the alkyl chain. Lowest toxicities were exhibited by compounds with halo substitutions. Among the halogenated compounds, dichlorinated compound exhibited the highest toxicity but less toxic as compared to alkoxy derivatives (Table 4). As per ECOSAR prediction, all the chemicals are inside AD and not missing predictions. It was observed that the acute as well as chronic toxicity values have negative linear correlations with the log K_OW_ values (Figures 7, 8).

**TABLE 4:** Baseline toxicity of the synthesized compounds.

Compounds	Molecular weight	LogKow (Kow Win estimate)	Water solubility (mg/L) (WsKow Win estimate)	LC_50_/EC_50_ (mg/L)			ChV (mg/L)		
				Fish	Daphnid	Green algae	Fish	Daphnid	Green algae
6a	278.3	3.71	14.143	0.190	1.60	0.208	0.032	0.028	0.114
6b	292.3	3.38	17.68	0.685	2.82	0.330	0.050	0.048	0.017
6c	306.32	3.87	5.57	0.235	1.50	0.205	0.031	0.025	0.114
6d	320.35	4.36	1.75	0.080	0.79	0.128	0.020	0.013	0.074
6e	320.35	4.29	2.03	0.095	0.878	0.138	0.021	0.015	0.079
6f	334.38	4.85	0.55	0.027	0.41	0.0793	0.012	0.007	0.048
6g	348.4	5.34	0.17	0.009	0.221	0.0492	0.007	0.003	0.031
6h	362.43	5.84	0.05	0.003	0.116	0.0304	0.005	0.002	0.020
6i	376.46	6.33	0.02	0.001	0.061	0.0188	0.003	0.001	0.013
6j	388.51	7.23	0.002	0.00015	0.018	0.0075	0.0003	0.0057	0.0011
6k	404.51	7.31	0	0.00013	0.016	0.007	0.001	0.0003	0.005
6l	418.54	7.8	0	0.00004	0.008	0.004	0.0008	0.0002	0.003
6m	446.59	8.78	0	0.000004	0.002	0.001	0.0003	0.00004	0.001
6n	460.62	9.27	0	0.000001	0.001	0.001	0.0002	0.00002	0.0009
6o	296.71	3.94	5.5	0.193	1.31	0.184	0.028	0.022	0.010
6p	331.16	4.59	0.97	0.049	0.59	0.104	0.016	0.010	0.061
6q	280.26	3.5	16.39	0.5	2.29	0.279	0.042	0.039	0.150
6r	280.26	3.5	16.39	0.5	2.29	0.279	0.042	0.039	0.150
6s	341.17	4.19	1.85	0.12	1.07	0.163	0.025	0.018	0.093
6t	310.74	4.49	1.55	0.058	0.643	0.108	0.0170	0.011	0.063

## 4 Conclusion

In conclusion, a series of twenty compounds were synthesised and characterised successfully. Out of 20 compounds 19 were found novel (6b- 6t). All the synthesized compounds were found to have fungicidal activity against *S. rolfsii* and *F. oxysporum*. It was observed that the synthesised compounds have less effectiveness against *F. oxysporum*. Compound 6t and 6f were found to be very effective against *Sclerotium rolfsii.* Also, molecular docking studies support the *in vitro* bioassay result where compound 6t with highest negative binding energy value (−17.77 kcal mol^−1^) showed better binding with lanosterol enzyme. Therefore, these two compounds could be used for effective management of *Sclerotium rolfsii.*

## Data Availability

The original contributions presented in the study are included in the article/Supplementary Material, further inquiries can be directed to the corresponding author.
