# Supplementary material for: Novel indazolylchromones: synthesis, fungicidal evaluation, molecular docking and aquatic toxicity prediction
Source: Front Chem. 2024 Jun 11;12:1411187. doi: 10.3389/fchem.2024.1411187 (PMC11196782; doi:10.3389/fchem.2024.1411187)
Supplement: Supplementary file 1 [file DataSheet1.docx]

**Novel Indazolylchromones: Synthesis, fungicidal evaluation, molecular docking and aquatic toxicity prediction**

Riya Kundu^a,c^, Najam Akthar Shakil^a^, Neethu Narayanan^a^, Deeba Kamil^b^, Virendra Singh Rana^a^_,_ Kailash P. Tripathi and Parshant Kaushik ^a*^

*^a^Division of Agricultural Chemicals, ^b^Division of Plant Pathology ICAR-Indian Agricultural Research Institute, New Delhi- 110 012, India*

*^c^The Graduate School, ICAR-IARI*

*Corresponding author: E-mail address: [parshantagrico@gmail.com](mailto:parshantagrico@gmail.com)

**Supporting Information**

**Spectral analysis of synthesized compounds**

**2-Indazol-1-yl-7-hydroxy- chromen-4-one(6a)**

Yellowish white solid; m.p.: 127–128°C, R_f_: 0.47 (hexane: ethyl acetate, 70:30). HREIMS m/z calcd for C_16_H_10_N_2_O_3_[M+H]^+^ = 279.0764, found = 279.0771. ^1^ H NMR (400 MHz, CDCl_3_): δ 8.21(1H, s, H-3’), 8.15- 8.19 (2H, m, H-5 and H-4’), 7.68 (1H, d, J=8, H-7’), 7.51- 7.57 (2H, m, H-6 and H-8), 7.42 (1H, t, J= 8.4, H-6’), 7.31 (1H, t, J=8, H-5’), 6.85 (1H, s, H-3). ^13^C NMR (100.6 MHz, CDCl_3_): 177.81 (C-4), 165.30 (C- 7), 157.96 (C- 2), 154.03 (C-9), 140.32 (C- 3’), 138.56 (C- 8’), 133.57 (C- 6’), 129.19 (C- 5), 126.46 (C-9’), 125.87 (C-5’),123.88 (C- 4’), 121.80 (C- 7’), 117.22 (C- 10), 113.46 (C- 6), 108.07 (C- 8), 96.40 (C- 3). IR (cm^−1^): 1365 (C-N stretch indazole), 1534 (C=C pyrone ring stretch), 1627 (C=C aromatic stretch) and 1681 (carbonyl stretch).

**2-Indazol-1-yl-7-methoxy- chromen-4-one (6b)**

Light-yellow solid; m.p.: 145-150°C, R_f_: 0.42 (hexane: ethyl acetate, 70:30).

HREIMS m/z calcd for C_17_H_12_N_2_O_3_ [M+H]^+^ = 293.0921; found = 293.0917. ^1^ H NMR (400 MHz, CDCl_3_): δ 8.27 (1H, s, H-3’), 8.14- 8.17 (2H, m, H-4’and H-5), 7.81(1H, d, J=8, H-7’), 7.62 (1H, t, J=7.2, H-6’), 7.38 (1H, t, J=7.6, H-5’), 7.00- 7.03 (2H, m, H-6 and H-8), 6.83 (1H, s, H-3), 3.97(3H, s, H-1”). ^13^C NMR (100.6 MHz, CDCl_3_): 177.37 (C-4), 164.05 (C- 7), 157.58 (C- 2), 155.64 (C-9), 140.06 (C- 3’), 138.49 (C- 8’), 129.09 (C- 6’), 127.34 (C- 5), 126.37 (C-9’), 123.76 (C- 5’), 121.78 (C- 4’), 117.44 (C- 10), 114.31 (C-7’), 113.26 (C- 6), 100.33 (C- 8), 96.36 (C- 3), 55.99 (C- 1”). IR (cm^−1^): 1363 (C-N stretch indazole), 1532 (C=C pyrone ring stretch), 1625 (C= aromatic C stretch) and 1682 (carbonyl stretch).

**2-Indazol-1-yl-7-ethoxy chromen-4-one (6c)**

Light-yellow solid; m.p.: 180-185°C, R_f_: 0.44 (hexane: ethyl acetate, 70:30). HREIMS *m/z* calcd for C_18_H_14_N_2_O_3_ [M+H]^+^= 307.1077; found= 307.10. ^1^H NMR (400 MHz, CDCl_3_): δ 8.29 (1H, s, H-3’), 8.15- 8.26 ( 2H, m, H-5 and H-4’), 7.81 (1H, d, J=8, H-7’), 7.63 (1H, t, J=7.6, H-6’), 7.38 (1H, t, J=8, H-5’), 6.98- 7.03 (2H, m, H-6 and H-8), 6.84 (1H, s, H-3), 4.22 (2H, q, J= 7.2, H-1”), 1.59 (3H, t, J=6.8, H-2”). ^13^C NMR (100.6 MHz, CDCl_3_): 176.98 (C-4), 162.48 (C- 7), 157.93 (C- 2), 155.62 (C-9), 140.30 (C- 3’), 138.34 (C- 8’), 129.05 (C- 6’), 127.82 (C- 5), 126.36 (C-9’), 123.84 (C- 5’), 121.47 (C- 4’), 118.96 (C- 10), 114.80 (C-7’), 109.99 (C- 6), 100.85 (C- 8), 97.10 (C- 3), 65.89 (C- 1”), 14.63 (C-2”). IR (cm^−1^): 1365 (C-N stretch indazole), 1531 (C=C pyrone ring stretch), 1626 (C=C aromatic stretch) and 1679 (carbonyl stretch).

**2-Indazol-1-yl-7-propoxy chromen-4-one (6d)**

Yellowish white solid; m.p.: 138-145°C, R_f_: 0.45 (hexane: ethyl acetate,70:30). HREIMS *m/z* calcd for C_19_H_16_N_2_O_3_ [M+H]^+^*=* 321.1234; found= 321.1219. ^1^ H NMR (400 MHz, CDCl_3_): δ 8.29 (1H, s, H-3’), 8.16- 8.20 ( 2H, m, H-5 and H-4’), 7.82 (1H, d, J=7.2, H-7’), 7.62 (1H, t, J=7.2, H-6’), 7.39 (1H, t, J= 7.2, H-5’), 7.02- 7.04 (2H, m, H-6 and H-8), 6.85 (1H, s , H-3), 4.09 (2H, t, J= 6.4, H-1”), 1.22-1.89 (2H, m, H-2”), 1.10 (3H, t, J= 6.8, H-3”). ^13^C NMR (100.6 MHz, CDCl_3_): 178.03 ( C-4), 163.67 ( C- 7), 157.68 ( C- 2), 155.62 ( C-9), 138.64 (C- 8’), 129.06 (C- 6’), 127.32 (C- 5), 126.81 ( C-9’), 123.74 (C- 5’), 121.79 (C- 4’), 120.96 ( C- 10), 114.58 ( C-7’), 113.29 ( C- 6), 100.89 ( C- 8), 96.42 ( C- 3), 70.32 ( C- 1”), 22.38 (C- 2”), 10.47 ( C- 3”). IR (cm^−1^): 1368 (C-N stretch indazole), 1535 (C=C pyrone ring stretch), 1627 (C=C aromatic stretch) and 1677 (carbonyl stretch).

**2-Indazol-1-yl-7-isopropoxy chromen-4-one (6e)**

Yellowish white solid; m.p.: 195-200°C, R_f_: 0.46 (hexane: ethyl acetate, 70:30). HREIMS *m/z* calcd for C_19_H_16_N_2_O_3_ [M+H]^+^*=* 321.1234; found= 321.1229. ^1^ H NMR (400 MHz, CDCl_3_): δ 8.27 (1H, s, H-3’), 8.12- 8.20 ( 2H, m, H-5 and H-4’), 7.89 (1H, d, J=7.2, H-7’), 7.60 (1H, t, J=7.2, H-6’), 7.38 (1H, t, J= 7.2, H-5’), 6.96- 7.01 (2H, m, H-6 and H-8), 6.87 (1H, s , H-3), 4.65- 4.68 (1H, m, J= 6.8, H-1”), 1.46 (1H, d, J= 6.0, CH_3_). ^13^C NMR (100.6 MHz, CDCl_3_): 178.05 (C-4), 163.68 (C- 7), 157.90 (C- 2), 155.61 (C-9), 140.05 (C- 3’), 138.63 (C- 8’), 129.05 (C- 6’), 127.33 (C- 5), 126.82 (C-9’), 123.79 (C- 5’), 121.74 (C- 4’), 120.96 (C- 10), 116.45 (C-7’), 115.24 (C- 6), 101.33 (C- 8), 96.43 (C- 3), 70.86 (C- 1”), 21.70 (i- CH_3_). IR (cm^−1^): IR (cm^−1^): 1370 (C-N stretch indazole), 1533 (C=C pyrone ring stretch), 1626 (C=C aromatic stretch) and 1678 (carbonyl stretch).

**2-Indazol-1-yl-7-butoxy chromen-4-one (6f)**

Yellowish white solid; m.p.: 135-140°C, R_f_: 0.47 (hexane: ethyl acetate, 70:30). HREIMS *m/z* calcd for C_20_H_18_N_2_O_3_ [M+H]^+^= 335.1390; found= 335.1381. ^1^ H NMR (400 MHz, CDCl_3_): δ 8.27 (1H, s, H-3’), 8.13- 8.18 ( 2H, m, H-5 and H-4’), 7.80 (1H, d, J=8, H-7’), 7.59 (1H, t, J=7.2, H-6’), 7.35 (1H, t, J=7.6, H-5’), 6.97- 7.01 (2H, m, H-6 and H-8), 6.83 (1H, s , H-3), 4.12 (2H, t, J= 6.8, H-1”), 1.54-1.88 (4H, m, H-2” and H-3”), 1.05 (3H, t, J = 7.2 , H-4”). ^13^C NMR (100.6 MHz, CDCl_3_): 177.42 (C-4), 163.65 (C- 7), 157.56 (C- 2), 155.62 (C-9), 140.02 (C- 3’), 138.47 (C- 8’), 129.05 (C- 6’), 127.25 (C- 5), 126.34 ( C-9’), 123.73 (C- 5’), 121.75 (C- 4’), 117.27 (C- 10), 114.56 (C-7’), 113.29 (C- 6), 100.82 (C- 8), 96.28 (C- 3), 68.53 (C- 1”), 31.01 (C- 2”), 19.27(C- 3”), 13.79 (C-4”). IR (cm^−1^): 1368 (C-N stretch indazole), 1525 (C=C pyrone ring stretch), 1631 (C=C aromatic stretch) and 1680 (carbonyl stretch).

**2-Indazol-1-yl-7-pentyloxy chromen-4-one (6g)**

Yellowish white solid; m.p.: 143- 148°C, R_f_: 0.44 (hexane: ethyl acetate, 70:30). HREIMS *m/z* calcd for C_21_H_20_N_2_O_3_ [M+H]^+^= 349.1547; found= 349.1539. ^1^ H NMR (400 MHz, CDCl_3_): 8.29 (1H, s, H-3’), 8.16- 8.20 (2H, m, H-5 and H-4’), 7.83 (1H, d, J=7.2, H-7’), 7.62 (1H, t, J=7.2, H-6’), 7.39 (1H, t, J=7.2, H-5’), 6.99- 7.02 (2H, m, H-6 and H-8), 6.86 (1H, s , H-3), 4.76 (2H, t, J= 6, H-1”), 1.38-1.60 (6H, m, H-2”- H- 4”), 1.28 (3H, t, J = 5.2, H-5”). ^13^C NMR (100.6 MHz, CDCl_3_): 177.47 ( C-4), 162.57 ( C- 7), 157.88 ( C- 2), 155.27 ( C-9), 140.07 ( C- 3’), 138.56 (C- 8’), 129.08 (C- 6’), 127.42 (C- 5), 126.40 ( C-9’), 123.77 (C- 5’), 121.81 (C- 4’), 117.10 ( C- 10), 115.14 ( C-7’), 113.28 ( C- 6), 102.04 ( C- 8), 96.41 ( C- 3), 70.91 ( C- 1”), 31.75 (C- 2”), 27.92 ( C- 3”), 21.87 ( C- 4”), 14.88 (C-5”). IR (cm^−1^): 1366 (C-N stretch indazole), 1529 (C=C pyrone ring stretch), 1629 (C=C aromatic stretch) and 1672 (carbonyl stretch).

**2-Indazol-1-yl-7-hexyloxy chromen-4-one (6h)**

Yellowish white solid; m.p.: 125-130°C, R_f_: 0.46 (hexane: ethyl acetate, 70:30). HREIMS *m/z* calcd for C_22_H_22_N_2_O_3_ [M+H]^+^= 363.1703; found= 363.1711. ^1^ H NMR (400 MHz, CDCl_3_): δ 8.28 (1H, s, H-3’), 8.15- 8.20 ( 2H, m, H-5 and H-4’), 7.82 (1H, d, J=8, H-7’), 7.62 (1H, t, J=7.2, H-6’), 7.37 (1H, t, J= 7.2, H-5’), 7.01- 7.03 (2H, m, H-6 and H-8), 6.85 (1H, s , H-3), 4.20 (2H, t, J= 6.8, H-1”), 1.37-1.88 (8H, m, H-2”- H-5”), 0.93 (3H, t, J= 7.2, H-6”). ^13^C NMR (100.6 MHz, CDCl_3_): 177.47 ( C-4), 163.69 ( C- 7), 157.60 ( C- 2), 155.68 ( C-9), 140.05 ( C- 3’), 138.54 (C- 8’), 129.07 (C- 6’), 127.31 (C- 5), 126.38 ( C-9’), 123.75 (C- 5’), 121.79 (C- 4’), 117.26 ( C- 10), 114.58 ( C-7’), 113.30 ( C- 6), 100.88 ( C- 8), 96.38 ( C- 3), 68.87 ( C- 1”), 31.50 (C- 2”), 28.97 ( C- 3”), 25.64 ( C- 4”), 22.56 (C-5”), 14.01 (C-6”). IR (cm^−1^): 1368 (C-N stretch indazole), 1531 (C=C pyrone ring stretch), 1628 (C=C aromatic stretch) and 1676 (carbonyl stretch).

**2-Indazol-1-yl-7-heptyloxy chromen-4-one (6i)**

Brownish yellow solid; m.p.: 115-120°C, R_f_: 0.47 (hexane: ethyl acetate, 70:30). HREIMS *m/z* calcd for C_23_H_24_N_2_O_3_ [M+H]^+^= 377.1860; found= 377.1855.^1^ H NMR (400 MHz, CDCl_3_): δ 8.27 (1H, s, H-3’), 8.15- 8.17( 2H, d, H-5 and H-4’), 7.81(1H, d, J=8, H-7’), 7.61(1H, t, J=7.2, H-6’), 7.35 (1H, t, J= 7.2, H-5’), 7.00- 7.02 (2H, m, H-6 and H-8), 6.84 (1H, s , H-3), 4.11 (2H, t, J= 6.8, H-1”), 1.32-1.86 (10H, m, H-2”- H-6”), 0.92 (3H, t, J = 6.8, H-7”). ^13^C NMR (100.6 MHz, CDCl_3_): 177.52 ( C-4), 163.66 ( C- 7), 157.57 ( C- 2), 155.66 ( C-9), 140.01 ( C- 3’), 138.51 (C- 8’), 129.05 (C- 6’), 127.28 (C- 5), 126.37 ( C-9’), 123.73 (C- 5’), 121.76 (C- 4’), 120.95 ( C- 10), 114.57 ( C-7’), 113.29 ( C- 6), 100.76 ( C- 8), 96.36 ( C- 3), 68.85 ( C- 1”), 31.71 (C-2”), 29.65 (C-3”), 28.98 (C- 4”), 25.91 ( C- 5”), 22.57 ( C- 6”), 14.06 ( C- 7”). IR (cm^−1^): 1365 (C-N stretch indazole), 1531 (C=C pyrone ring stretch), 1625 (C=C aromatic stretch) and 1675 (carbonyl stretch).

**2-Indazol-1-yl-7-octyloxy chromen-4-one (6j)**

Brownish yellow solid; m.p.: 145-150°C, R_f_: 0.48 (hexane: ethyl acetate, 70: 30). HREIMS *m/z* calcd for C_24_H_26_N_2_O_3_ [M+H]^+^*=* 391.2016; found= 391.2023. ^1^ H NMR (400 MHz, CDCl_3_): δ 8.27 (1H, s, H-3’), 8.13- 8.17 ( 2H, m, H-5 and H-4’), 7.78 (1H, d, J=8, H-7’), 7.59 (1H, t, J=7.2, H-6’), 7.35 (1H, t, J=7.2, H-5’), 6.98- 7.01 (2H, m, H-6 and H-8), 6.82 (1H, s , H-3), 4.11 (2H, t, J= 6.8, H-1”), 1.30-1.89 (12H, m, H-2”- H- 7”), 0.91 (3H, t, J = 6.8, H-8”). ^13^C NMR (100.6 MHz, CDCl_3_): 177.41 ( C-4), 163.63 ( C- 7), 157.54 ( C- 2), 155.62 ( C-9), 139.98 ( C- 3’), 138.48 (C- 8’), 129.03 (C- 6’), 127.24 (C- 5), 126.34 ( C-9’), 123.71 (C- 5’), 121.73 (C- 4’), 117.26 ( C- 10), 114.53 ( C-7’), 113.28 ( C- 6), 100.82 ( C- 8), 96.31 ( C- 3), 68.85 ( C- 1”), 31.76 (C-2”), 29.27 (C-3”), 29.18 (C-4”), 28.99 (C- 5”), 25.94 ( C- 6”), 22.61 ( C- 7”), 14.07 ( C- 8”). IR (cm^−1^): 1365 (C-N stretch indazole), 1532 (C=C pyrone ring stretch), 1624 (C=C aromatic stretch) and 1677 (carbonyl stretch).

**2-Indazol-1-yl-7-nonyloxy chromen-4-one (6k)**

Yellow solid; m.p.: 100-105°C, R_f_: 0.48 (hexane: ethyl acetate, 70: 30). HREIMS *m/z* calcd for C_25_H_28_N_2_O_3_ [M+H]^+^= 405.2173; found= 405.2156. ^1^ H NMR (400 MHz, CDCl_3_): δ 8.28 (1H, s, H-3’), 8.15- 8.19 ( 2H, m, H-5 and H-4’), 7.81(1H, d, J= 8, H-7’), 7.60 (1H, t, J= 7.2, H-6’), 7.36 (1H, t, J= 7.2, H-5’), 7.00- 7.02 (2H, m, H-6 and H-8), 6.84 (1H, s , H-3), 4.12 (2H, t, J= 7.2, H-1”), 1.28-1.88 (14H, m, H-2”- H-8”), 0.89 (3H, t, J = 7.2, H-9”). ^13^C NMR (100.6 MHz, CDCl_3_): 177.51 ( C-4), 163.67 ( C- 7), 157.58 ( C- 2), 155.66 ( C-9), 140.02 ( C- 3’), 138.52 (C- 8’), 129.05 (C- 6’), 127.29 (C- 5), 126.37 ( C-9’), 123.73 (C- 5’), 121.77 (C- 4’), 117.28 ( C- 10), 114.56 ( C-7’), 113.29 ( C- 6), 100.87 ( C- 8), 96.37 ( C- 3), 68.86 ( C- 1”), 31.83 (C-2”), 29.48 (C-3”), 29.32 (C-4”), 29.22 (C- 5”), 29.00 ( C- 6”), 25.95 ( C- 7”), 22.63 ( C- 8”), 14.08 (C-9”). IR (cm^−1^): 1369 (C-N stretch indazole), 1531 (C=C pyrone ring stretch), 1626 (C=C aromatic stretch) and 1676 (carbonyl stretch).

**2-Indazol-1-yl-7 -decyloxy chromen-4-one (6l)**

Yellowish white solid; m.p.: 85-90°C, R_f_: 0.45 (hexane: ethyl acetate, 70: 30). HREIMS *m/z* calcd for C_26_H_30_N_2_O_3_ [M+H]^+^ = 419.2329; found= 419.2322. ^1^ H NMR (400 MHz, CDCl_3_): δ 8.28 (1H, s, H-3’), 8.11- 8.17 ( 2H, m, H-5 and H-4’), 7.81 (1H, d, J= 8, H-7’), 7.51 (1H, t, J= 6.8, H-6’), 7.39 (1H, t, J= 7.2, H-5’), 7.00- 7.03 (2H, m, H-6 and H-8), 6.87 (1H, s , H-3), 4.11 (2H, t, J= 7.2, H-1”), 1.28-1.85 (16H, m, H-2”- H-9”), 0.88 (3H, t, J = 7.2, H-10”). ^13^C NMR (100.6 MHz, CDCl_3_): 177.79 ( C-4), 163.70 ( C- 7), 157.48 ( C- 2), 155.59 ( C-9), 140.01 ( C- 3’), 138.21 (C- 8’), 129.06 (C- 6’), 127.30 (C- 5), 126.81 ( C-9’), 123.74 (C- 5’), 121.79 (C- 4’), 120.84 ( C- 10), 114.61 ( C-7’), 113.28 ( C- 6), 100.89 ( C- 8), 96.40 ( C- 3), 68.85 ( C- 1”), 31.83 (C-2”), 29.98 (C-3”), 29.76 (C-4”), 29.51 (C-5”), 29.27 (C- 6”), 28.99 ( C- 7”), 25.94 ( C- 8”), 22.64 ( C- 9”), 14.08 (C-10”). IR (cm^−1^): 1365 (C-N stretch indazole), 1532 (C=C pyrone ring stretch), 1631 (C=C aromatic stretch) and 1675 (carbonyl stretch).

**2-Indazol-1-yl-7-dodecyloxy chromen-4-one (6m)**

Yellow solid; m.p.: 78-85°C, R_f_: 0.45 (hexane: ethyl acetate, 70: 30). HREIMS *m/z* calcd for C_28_H_34_N_2_O_3_ [M+H]^+^= 447.2642; found= 447.2649. ^1^ H NMR (400 MHz, CDCl_3_): δ 8.29 (1H, s, H-3’), 8.15- 8.20 ( 2H, m, H-5 and H-4’), 7.89 ( 1H, d, J=8.4, H-7’), 7.60 (1H, t, J= 7.2, H-6’), 7.41 (1H, t, J=7.2, H-5’), 7.16- 7.20 (2H, m, H-6 and H-8), 6.85 (1H, s , H-3), 4.12 (2H, t, J= 7.2, H-1”), 1.25-1.95 (20H, m, H-2”- H-11”), 0.89 (3H, t, J = 7.2, H-9”). ^13^C NMR (100.6 MHz, CDCl_3_): 177.73 ( C-4), 163.43 ( C- 7), 158.20 ( C- 2), 154.84 ( C-9), 139.71 ( C- 3’), 137.66 (C- 8’), 129.47 (C- 6’), 127.01 (C- 5), 126.39 ( C-9’), 123.86 (C- 5’), 121.18 (C- 4’), 120.91 ( C- 10), 114.59 ( C-7’), 113.43 ( C- 6), 100.91 ( C- 8), 96.59 ( C- 3), 68.85 ( C- 1”), 33.79 (C-2”), 31.88 (C-3”), 29.66 (C-4”), 27.16 (C- 5”), 26.89 (C-6”), 26.73 ( C- 7”), 25.80 ( C- 8”), 24.66 ( C-9”), 23.58 ( C- 10”), 22.45 ( C- 11”), 14.10 ( C- 12”). IR (cm^−1^): 1367 (C-N stretch indazole), 1534 (C=C pyrone ring stretch), 1629 (C=C aromatic stretch) and 1680 (carbonyl stretch).

**2-Indazol-1-yl-7 -tridecyloxychromen-4-one (6n)**

Yellow solid; m.p.: 120-125°C, R_f_: 0.48 (hexane: ethyl acetate, 70: 30). HREIMS *m/z* calcd for C_29_H_36_N_2_O_3_ [M+H]^+^= 461.2799; found= 461.2784.^1^ H NMR (400 MHz, CDCl_3_): δ 8.28 (1H, s, H-3’), 8.06- 8.17 ( 2H, m, H-5 and H-4’), 7.78 (1H, d, J= 8, H-7’), 7.62 (1H, t, J= 7.4, H-6’), 7.39 (1H, t, J= 7.2, H-5’), 7.15- 7.19 (2H, m, H-6 and H-8), 6.82 (1H, s , H-3), 4.21 (2H, t, J= 7.2, H-1”), 1.21-1.87 (22H, m, H-2”- H-12”), 0.82 (3H, t, J = 6.8, H-13”). ^13^C NMR (100.6 MHz, CDCl_3_): 177.17 ( C-4), 163.70 ( C- 7), 157.58 ( C- 2), 155.86 ( C-9), 140.02 ( C- 3’), 138.14 (C- 8’), 129.56 (C- 6’), 127.45 (C- 5), 126.84 ( C-9’), 123.86 (C- 5’), 121.80 (C- 4’), 118.80 ( C- 10), 114.61 ( C-7’), 113.29 ( C- 6), 100.90 ( C- 8), 96.43 ( C- 3), 68.68 ( C- 1”), 33.79 (C-2”), 31.88 (C-3”), 29.66 (C-4”), 29.53 (C-5”), 29.33 (C-6”), 27.45 (C-7”), 27.06 (C- 8”), 26.85 (C-9”), 26.79 ( C- 10”), 25.84 ( C- 11”), 22.66 ( C- 12”), 14.09 (C-13”). IR (cm^−1^): 1364 (C-N stretch indazole), 1531 (C=C pyrone ring stretch), 1626 (C=C aromatic stretch) and 1679 (carbonyl stretch).

**2-Indazol-1-yl-6 -chloro chromen-4-one (6o)**

White solid; m.p.: 175-180°C, R_f_: 0.44 (hexane: ethyl acetate, 70: 30 ). HREIMS *m/z* calcd for C_16_H_9_ClN_2_O_2_ [M+H]^+^= 297.0425; found= 297.0431. ^1^H NMR (400 MHz, CDCl_3_): δ 8.30 (1H, s, H-3’), 8.18-8.22 (2H, m, H-5 & H-4’), 7.83 (1H, d, J=8 , H-7’), 7.69 (1H, dd, J= 8.8 & 2.4, H-7), 7.59-7.64 ( 2H, m, H-6’ & H-8), 7.41 (1H, t, J=7.2, H-5’), 6.94 (1H, s , H-3). ^13^C NMR (100.6 MHz, CDCl_3_): 176.35 (C-4), 158.04 (C- 2), 140.62 (C-3’), 138.41 (C-8’), 133.62 (C-7), 131.84 (C-6), 129.32 (C-5), 126.47 (C-9’), 125.58 (C-6’), 124.85 (C-10), 124.06 (C-4’), 121.87 (C-5’), 118.81 (C-8), 113.36 (C-7’), 96.12 (C-3). . IR (cm^-1^): 1367 (C-N stretch indazole), 1532 (C=C pyrone ring stretch), 1626 (C=C aromatic stretch) and 1677 (carbonyl stretch).

**2-Indazol-1-yl-6,8 -dichloro chromen-4-one (6p)**

Yellowish white solid; m.p.: 225-230°C, R_f_: 0.43 (hexane: ethyl acetate:, 70:30). HREIMS *m/z* calcd for C_16_H_8_ Cl_2_N_2_O_2_ [M+H]^+^*=* 331.0036; found= 331.0027. ^1^H NMR (400 MHz, CDCl_3_): δ 8.32 (1H, s, H-3’), 8.17 (1H, d, J= 2.4, H-4’), 7.83 (1H, d, J= 8, H-7’), 7.79 (1H, d, J= 2.4, H-7), 7.62- 7.67(2H, m, H-6’ and H-5), 7.40 (1H, t, J= 7.2, H-5’), 7.02 (1H, s, H-3). ^13^C NMR (100.6 MHz, CDCl_3_): 175.50 (C-4), 158.40 (C- 2), 149.90 (C-9), 141.19 (C- 3’), 138.44 (C- 8’), 133.58 (C-6), 131.67 (C- 8), 129.60 (C- 6’), 126.50 (C- 5), 126.05 (C-9’), 124.43 (C- 5’), 124.27 (C- 4’), 123.28 (C- 10), 121.78 (C-7), 113.82 (C-7’), 96.03 (C- 3). IR (cm^-1^): 1369 (C-N stretch indazole), 1529 (C=C pyrone ring stretch), 1630 (C=C aromatic stretch) and 1674 (carbonyl stretch).

**2-Indazol-1-yl-6 -flouro chromen-4-one (6q)**

Bright yellow solid; m.p.: 190-200°C, R_f_: 0.43 (hexane: ethyl acetate, 70: 30). HREIMS *m/z* calcd for C_16_H_9_ FN_2_O_2_ [M+H]^+^= 281.0721; found= 281.0729. ^1^ H NMR (400 MHz, CDCl_3_): δ8.30 (1H, s, H-3’), 8.22 (1H, d, J=8.8, H-4’), 7.93 (1H, dd, J=8 & 3.2, H-7), 7.83( 1H, d, J= 8, H-7’), 7.60- 7.67 (2H, m, H-5 and H-6’), 7.37- 7.48 (2H, m, H-8 and H-5’), 6.93 (1H, s , H-3). ^13^C NMR (100.6 MHz, CDCl_3_): 176.55 (C-4), 166.71 (C- 6), 158.32 (C- 2), 154.65 (C-9), 140.62 (C- 3’), 138.69 (C- 8’), 129.35 (C- 6’), 128.53 (C- 5), 126.72 (C-9’), 124.07 (C- 5’), 121.94 (C- 4’), 120.61 (C- 10), 114.59 (C-7), 113.33 (C-7’), 104.52 (C- 8), 96.36 (C- 3). IR (cm^−1^): 1367 (C-N stretch indazole), 1532 (C=C pyrone ring stretch), 1626 (C=C aromatic stretch) and 1676 (carbonyl stretch).

**2-Indazol-1-yl-7 -fluoro chromen-4-one (6r)**

Bright yellow solid; m.p.: 180-185°C, R_f_: 0.43 (hexane: ethyl acetate, 70: 30). HREIMS *m/z* calcd for C_16_H_9_ FN_2_O_2_ [M+H]^+^= 281.0721; found= 281.0738. ^1^ H NMR (400 MHz, CDCl_3_): δ 8.32 (1H, s, H-3’), 8.29 (1H, d, J= 8.8, H-4’), 8.19 ( 1H, d, J=8.8, H-5), 7.84 (1H, d, J= 8, H-7’), 7.65 (1H, t, J= 7.2, H-6’), 7.35-7.41 (2H, m, H-6 and H-8), 7.24 (1H, t, J= 7.2, H-5’), 6.93 (1H, s, H-3). ^13^C NMR (100.6 MHz, CDCl_3_): 176.96 (C-4), 166.76 (C- 7), 155.55 (C- 2), 154.45 ( C-9), 140.58 ( C- 3’), 138.82 (C- 8’), 134.82 (C- 6’), 129.31 (C- 5), 126.52 (C-9’), 124.04 (C- 5’), 121.91 (C- 4’), 120.96 (C- 10), 119.24 (C-7’), 113.39 (C- 6), 109.71 (C- 8), 95.83 (C- 3). IR (cm^−1^): 1365 (C-N stretch indazole), 15312 (C=C pyrone ring stretch), 1625 (C=C aromatic stretch) and 1678 (carbonyl stretch).

**2-Indazol-1-yl-6 -bromo chromen-4-one (6s)**

Yellow solid; m.p.: 170-173°C, R_f_: 0.45 (hexane: ethyl acetate, 70: 30). HREIMS *m/z* calcd for C_16_H_9_BrN_2_O_2_ [M+H]^+^= 340.9920; found= 340.9914.^1^ H NMR (400 MHz, CDCl_3_): δ 8.38 (1H, s, H-3’), 8.17-8.3 (2H, m, H-5& H-4’), 7.98 (1H, dd, J=8 & 3.2, H-7), 7.80 (1H, d, J=8.8, H-7’), 7.63 (1H, t, J= 7.2, H-6’), 7.54 (1H, d, J= 8.8, H-8), 7.38 (1H, t, J= 7.2, H-5’), 6.92 (1H, s , H-3). ^13^C NMR (100.6 MHz, CDCl_3_): 177.05 (C-4), 157.92 (C- 2), 152.58 (C-9), 140.67 (C- 3’), 138.48 (C- 8’), 136.46 (C- 6’), 128.82 (C- 5), 126.52 (C-9’), 124.09 (C- 5’), 125.307(C-6), 121.91 (C- 4’), 121.45 (C-10), 119.25 (C-7), 113.39 (C-7’), 111.53 (C- 8), 96.29 (C- 3). IR (cm^-1^): 1370 (C-N stretch indazole), 1530 (C=C pyrone ring stretch), 1629 (C=C aromatic stretch) and 1675 (carbonyl stretch).

**2-Indazol-1-yl-7-methyl-6-chlorochromen-4-one(6t)**

Creamish white solid; m.p.: 205-210°C, R_f_: 0.44 (hexane: ethyl acetate, 70: 30). HREIMS *m/z* calcd for C_17_H_11_ClN_2_O_2_ [M+H]^+^= 311.0582; found= 311.0573. ^1^H NMR (400 MHz, CDCl_3_): δ 8.29 (1H, s, H-3’), 8.16-8.21 (2H, m, H-5 and H-4’), 7.82 (1H, d, J= 8, H-7’), 7.64 (1H, t, J=8, H-6’), 7.54 (1H, s, H-8), 7.38 (1H, t, J=8, H-5’), 6.90 (1H, s, H-3), 2.56 (3H, s, H-Me). ^13^C NMR (100.6 MHz, CDCl_3_): 176.81 (C-4), 158.28 (C- 2), 152.11 (C-9), 142.84 (C- 7), 140.84 (C- 3’), 138.27 (C- 8’), 132.74 (C-6), 129.27 (C- 6’), 127.85 (C- 5), 126.50 (C-9’), 125.85 (C- 5’), 123.99 (C- 4’), 122.86 (C- 10), 121.68 (C-8), 119.07 (C-7’), 96.17 (C- 3), 29.67 (C-Me). IR (cm^-1^): 1368 (C-N stretch indazole), 1533 (C=C pyrone ring stretch), 1624 (C=C aromatic stretch) and 1678 (carbonyl stretch).

**Figure 1:** ^1^H NMR spectrum of compound **6a**

**Figure 2:** ^13^C NMR spectrum of compound **6a**

**Figure 3:** ^1^H NMR spectrum of compound **6b**

**Figure 4:** ^13^C NMR spectrum of compound **6b**

**Figure 5:** ^1^H NMR spectrum of compound **6c**

**Figure 6:** ^13^C NMR spectrum of compound **6c**

**Figure 7:** ^1^H NMR spectrum of compound **6d**

**Figure 8:** ^13^C NMR spectrum of compound **6d**

**Figure 9:** ^1^H NMR spectrum of compound **6e**

**Figure 10:** ^13^C NMR spectrum of compound **6e**

**Figure 11:** ^1^H NMR spectrum of compound **6f**

**Figure 12:** ^13^C NMR spectrum of compound **6f**

**Figure 13:** ^1^H NMR spectrum of compound **6g**

**Figure 14:** ^13^C NMR spectrum of compound **6g**

**Figure 15:** ^1^H NMR spectrum of compound **6h**

**Figure 16:** ^13^C NMR spectrum of compound **6h**

**Figure 17:** ^1^H NMR spectrum of compound **6i**

**Figure 18:** ^13^C NMR spectrum of compound **6i**

**Figure 19:** ^1^H NMR spectrum of compound **6j**

**Figure 20:** ^13^C NMR spectrum of compound **6j**

**Figure 21:** ^1^H NMR spectrum of compound **6k**

**Figure 22:** ^13^C NMR spectrum of compound **6k**

**Figure 23:** ^1^H NMR spectrum of compound **6l**

**Figure 24:** ^13^C NMR spectrum of compound **6l**

**Figure 25:** ^1^H NMR spectrum of compound **6m**

**Figure 26:** ^13^C NMR spectrum of compound **6m**

**Figure 27:** ^1^H NMR spectrum of compound **6n**

**Figure 28:** ^13^C NMR spectrum of compound **6n**

**Figure 29:** ^1^H NMR spectrum of compound **6o**

**Figure 30:** ^13^C NMR spectrum of compound **6o**

**Figure 31:** ^1^H NMR spectrum of compound **6p**

**Figure 32:** ^13^C NMR spectrum of compound **6p**

**Figure 33:** ^1^H NMR spectrum of compound **6q**

**Figure 34:** ^13^C NMR spectrum of compound **6q**

**Figure 35:** ^1^H NMR spectrum of compound **6r**

**Figure 36:** ^13^C NMR spectrum of compound **6r**

**Figure 37:** ^1^H NMR spectrum of compound **6s**

**Figure 38:** ^13^C NMR spectrum of compound **6s**

**Figure 39:** ^1^H NMR spectrum of compound **6t**

**Figure 40:** ^13^C NMR spectrum of compound **6t**

**Figure 41:** Representative IR of indazolylchromones


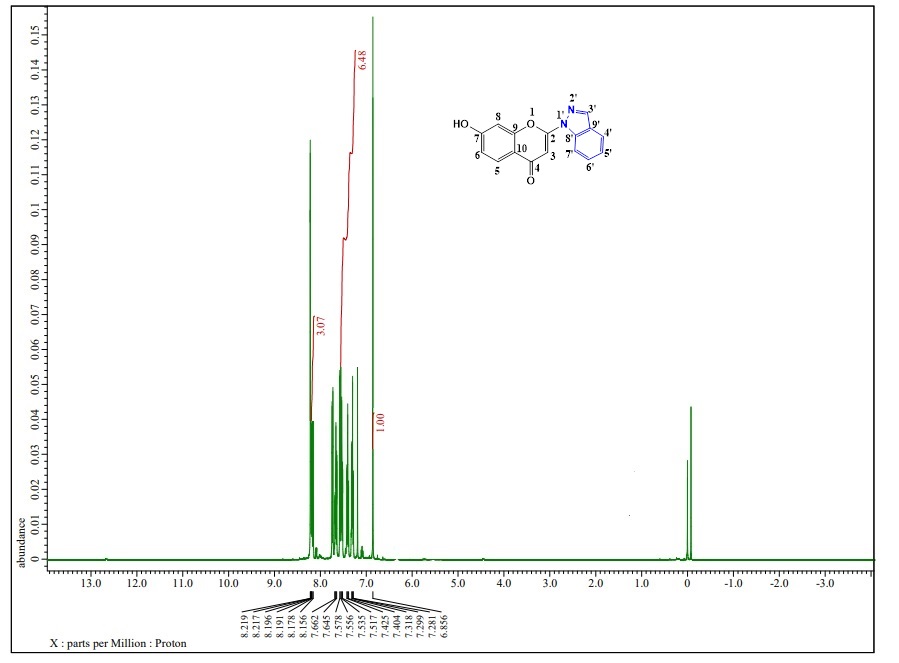


**Fig 1: ^1^H NMR of compound 6a**


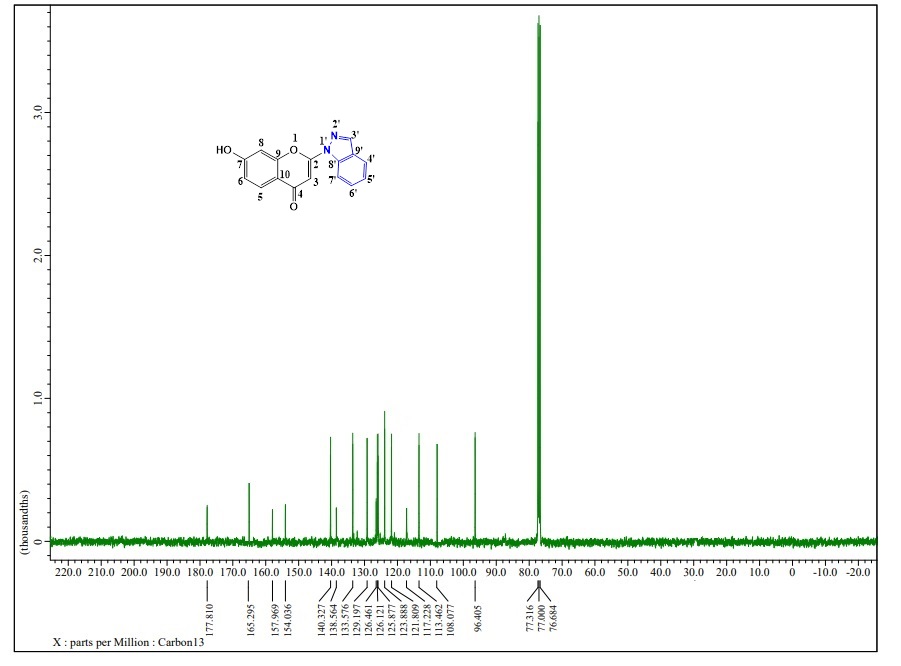


**Fig 2: ^13^C NMR of compound 6a**


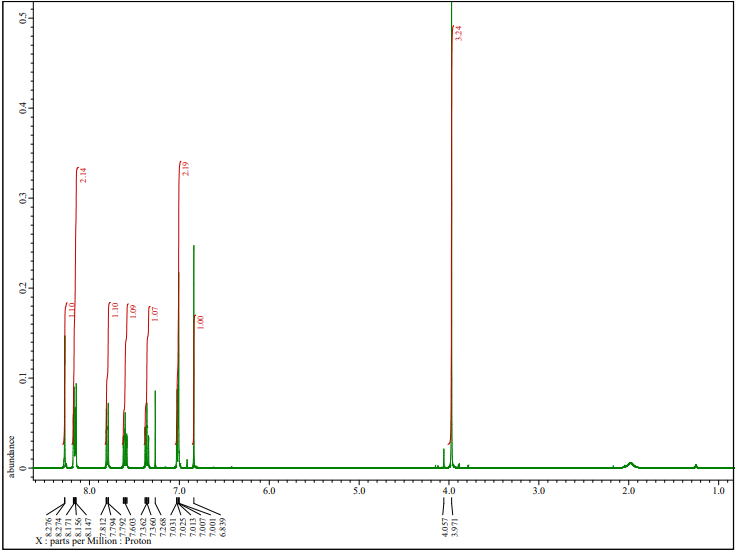


**Fig 3: ^1^H NMR of compound 6b**


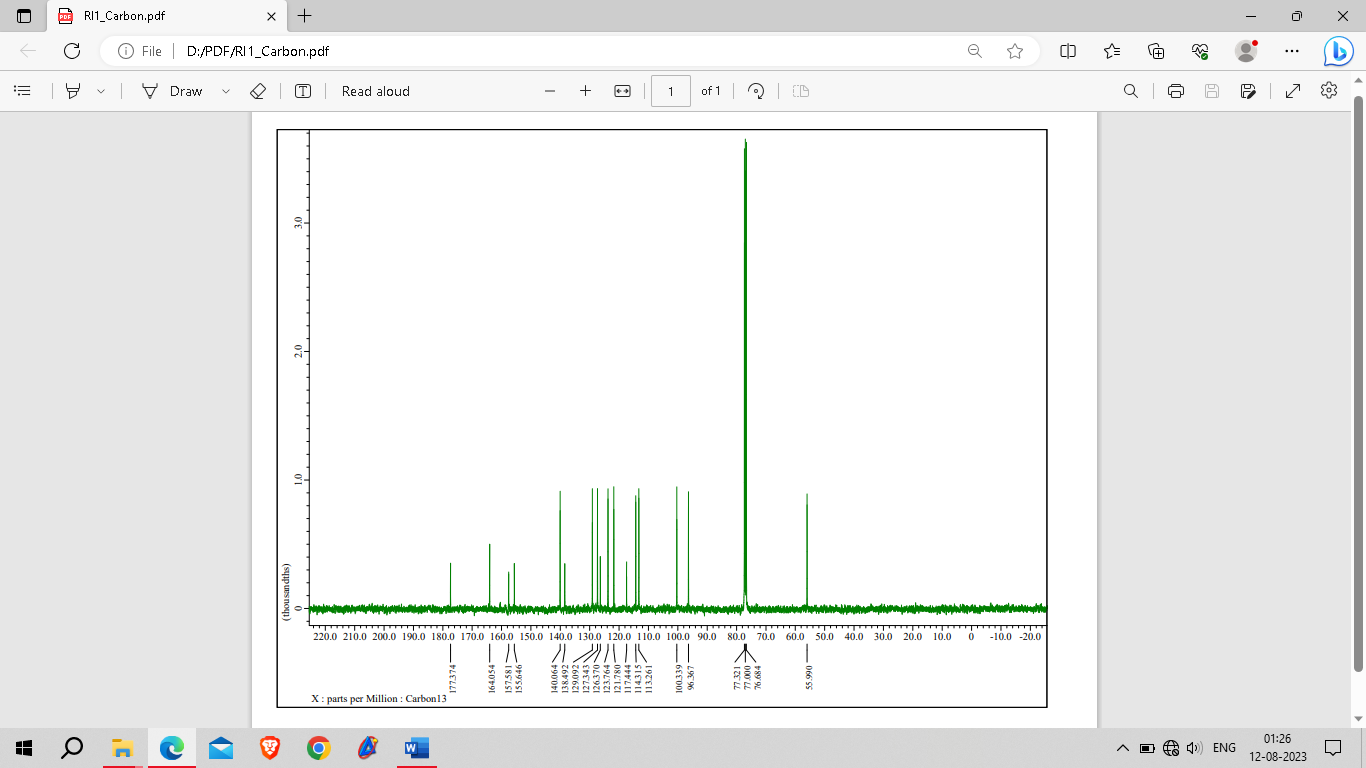


**Fig 4: ^13^C NMR of compound 6b**


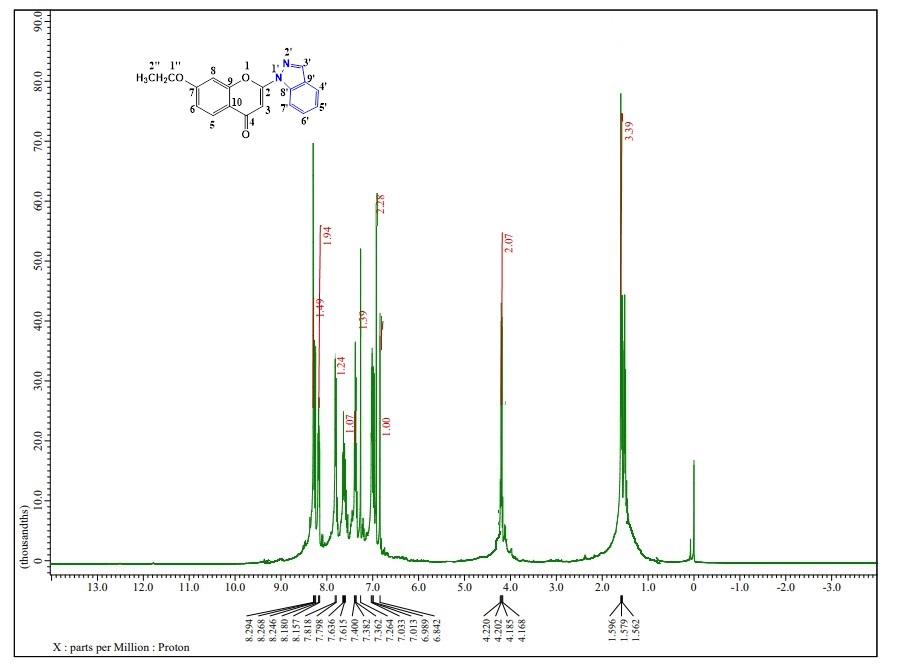


**Fig 5: ^1^H NMR of compound 6c**


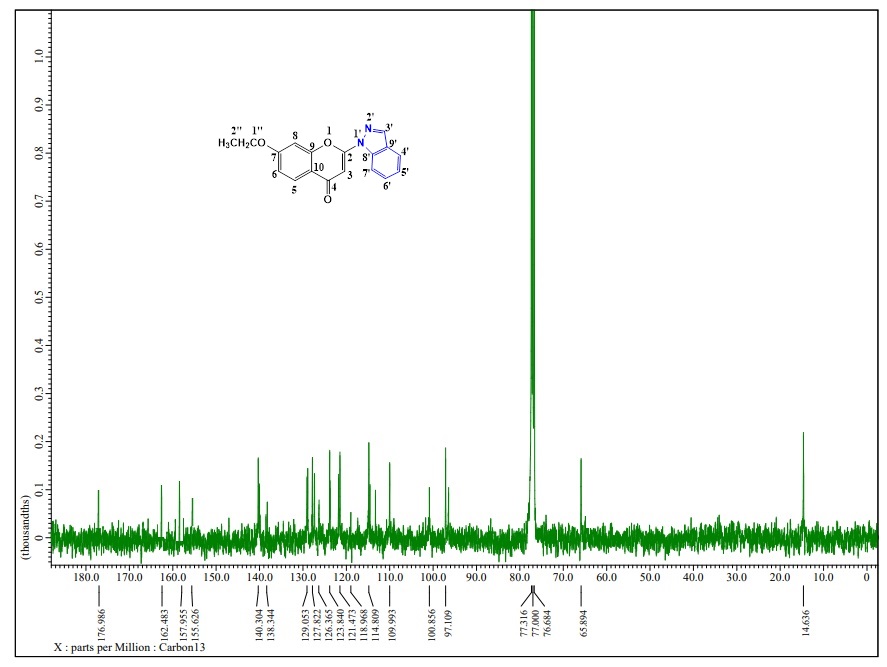


**Fig 6: ^13^C NMR of compound 6c**


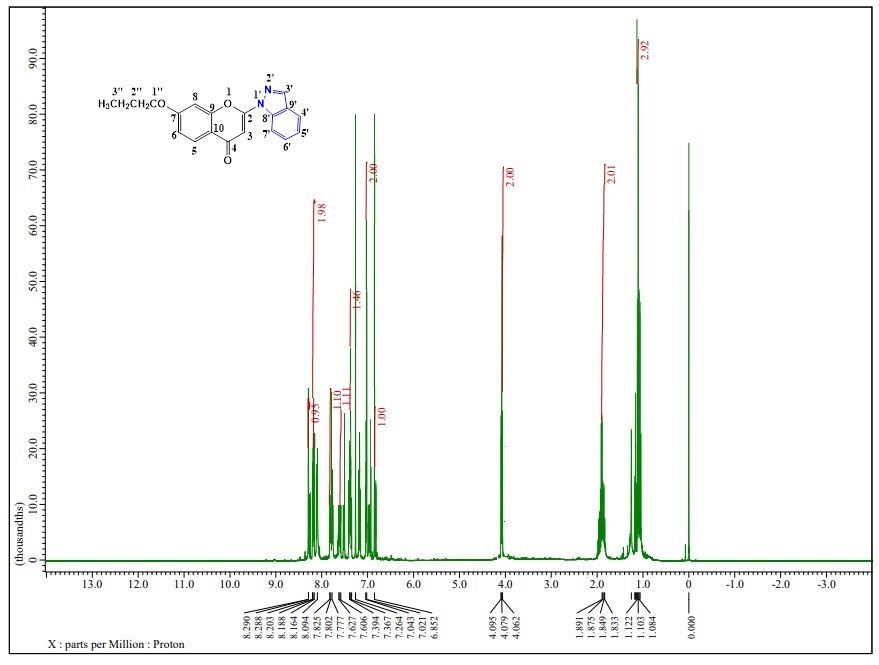


**Fig 7: ^1^H NMR of compound 6d**


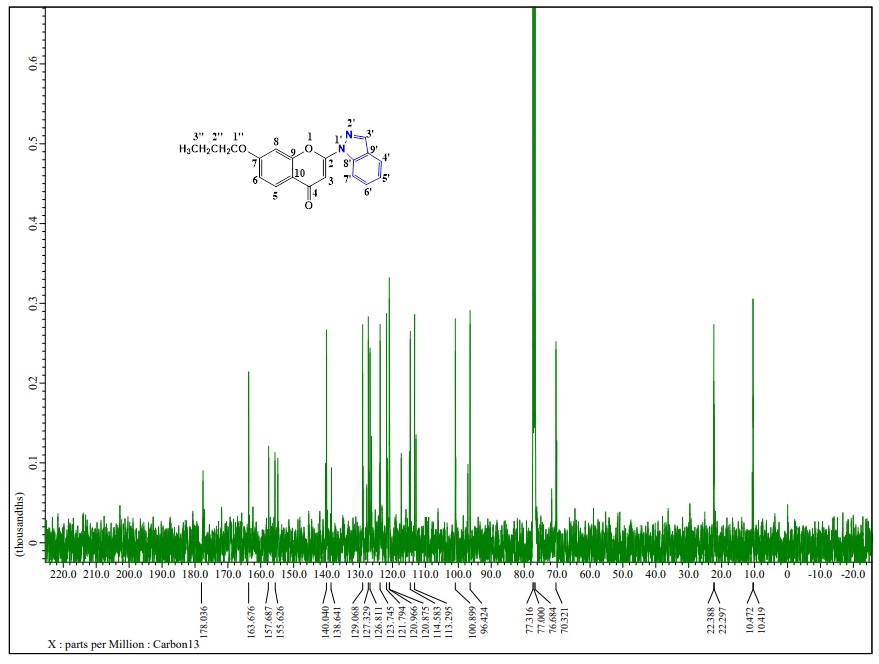


**Fig 8: ^13^C NMR of compound 6d**


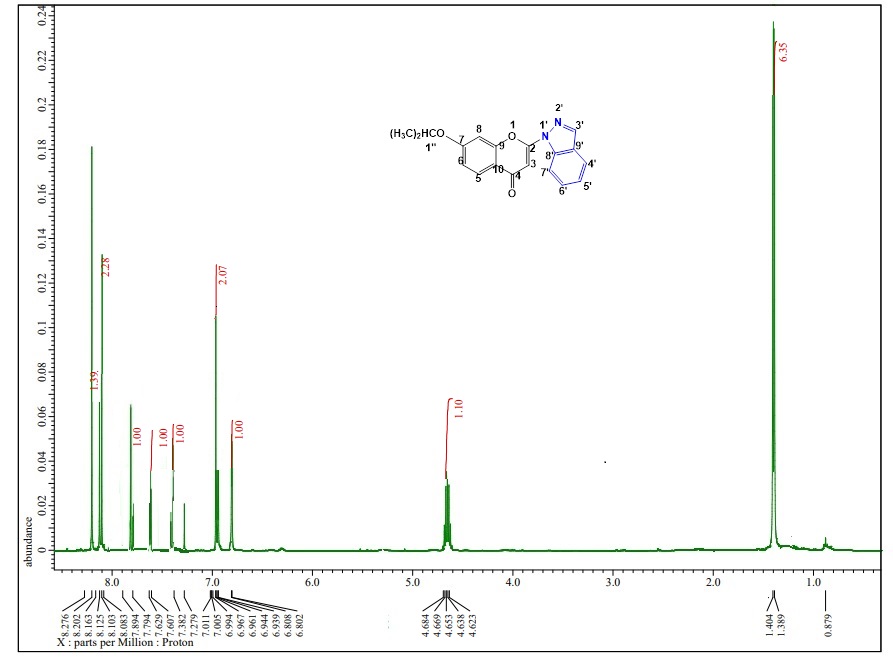


**Fig 9: ^1^H NMR of compound 6e**


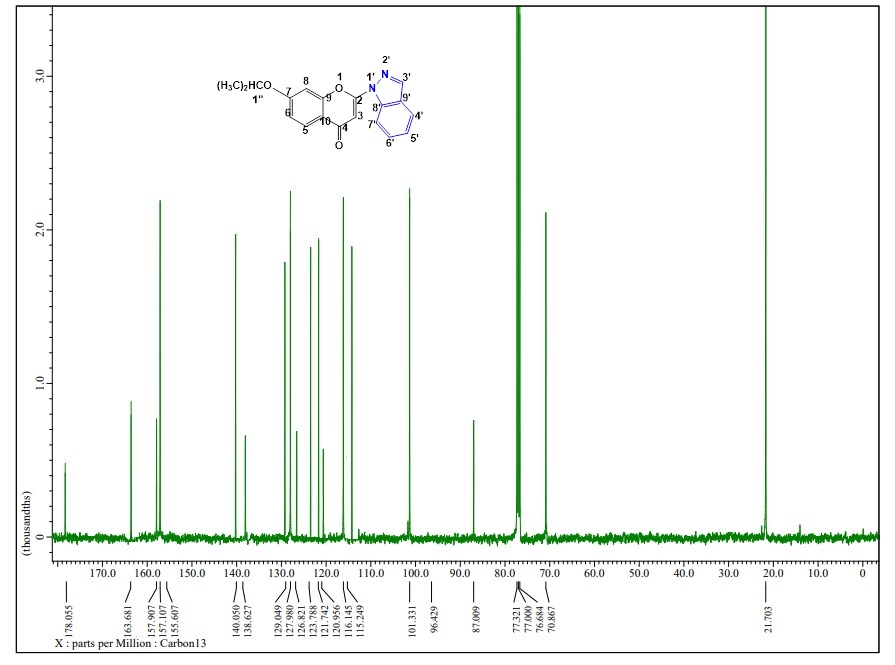


**Fig 10: ^13^C NMR of compound 6e**


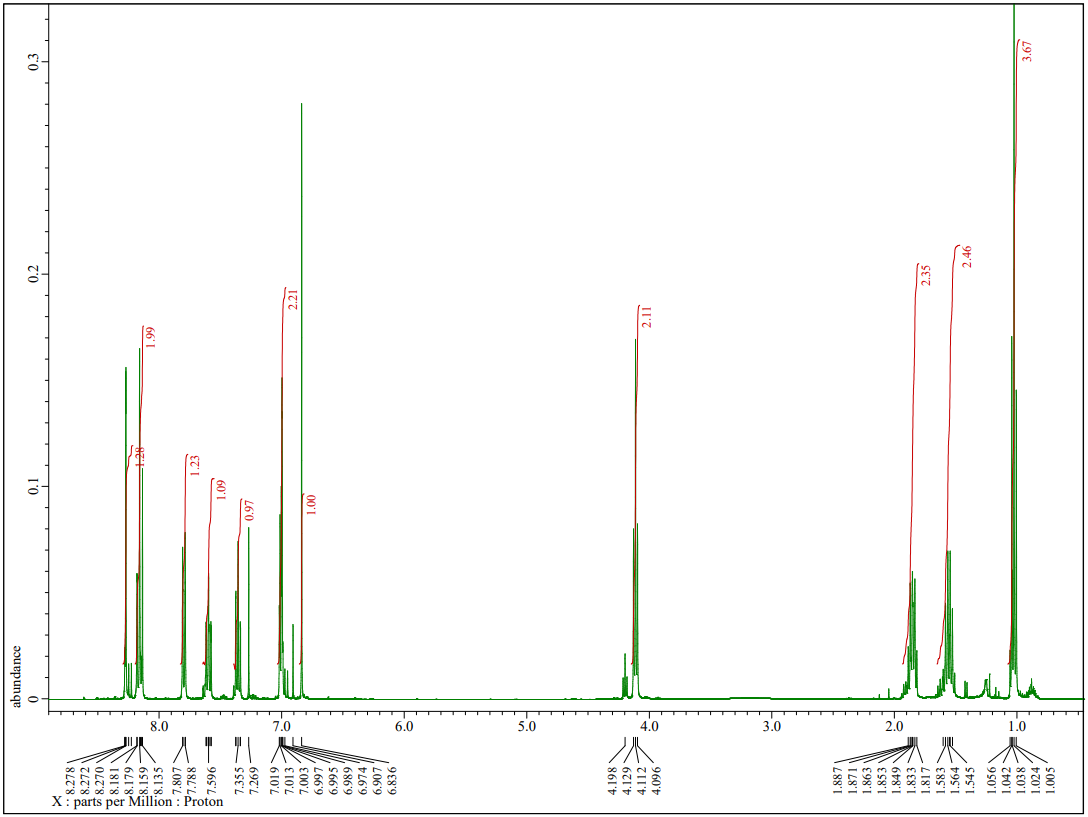


**Fig 11: ^1^H NMR of compound 6f**


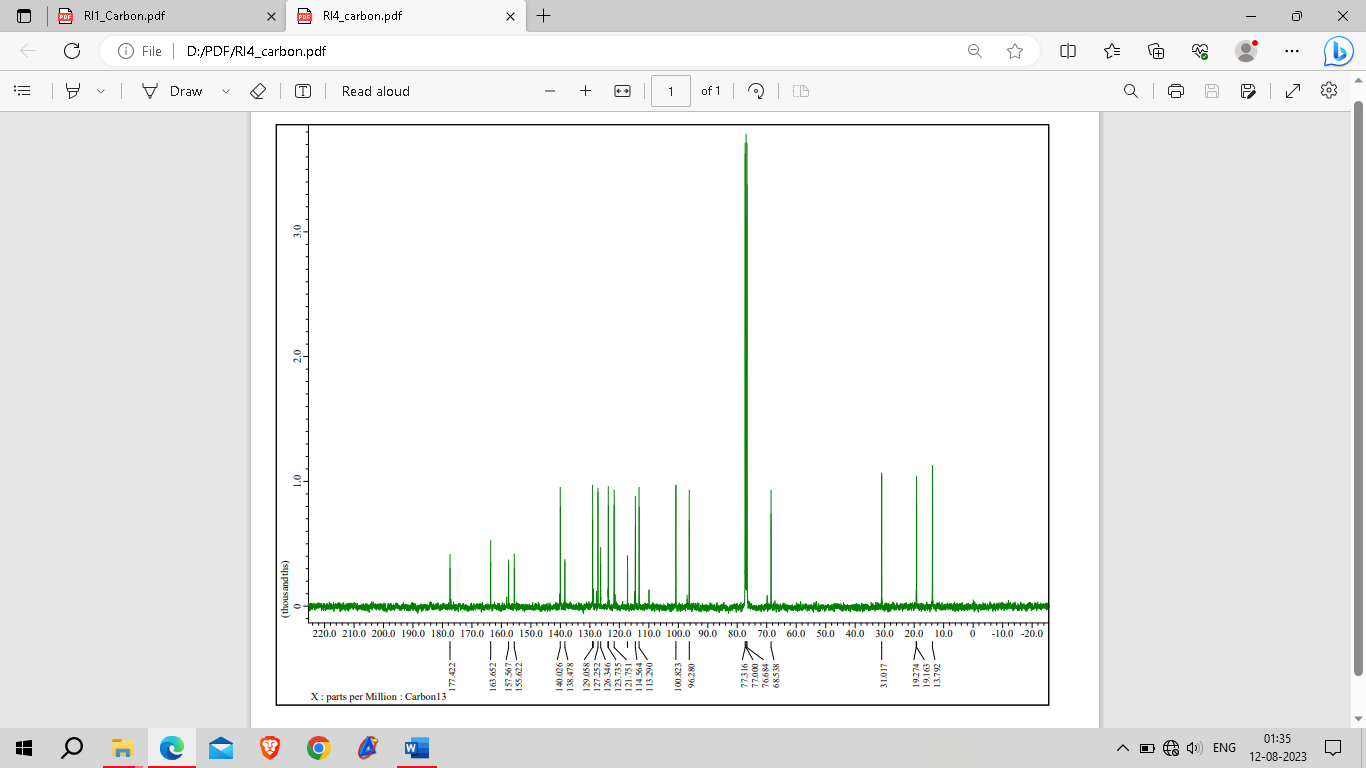


**Fig 12: ^13^C NMR of compound 6f**


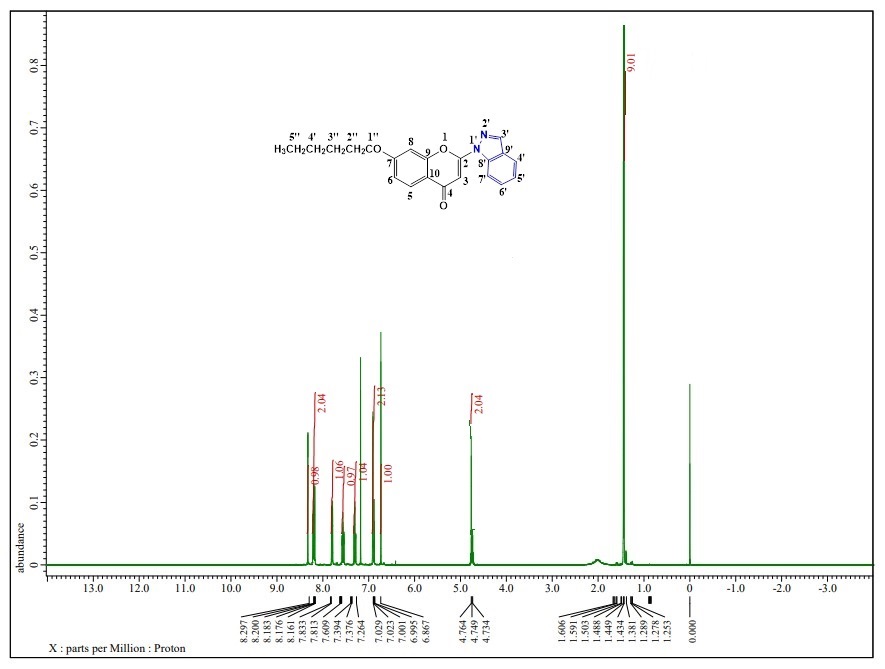


**Fig 13: ^1^H NMR of compound 6g**


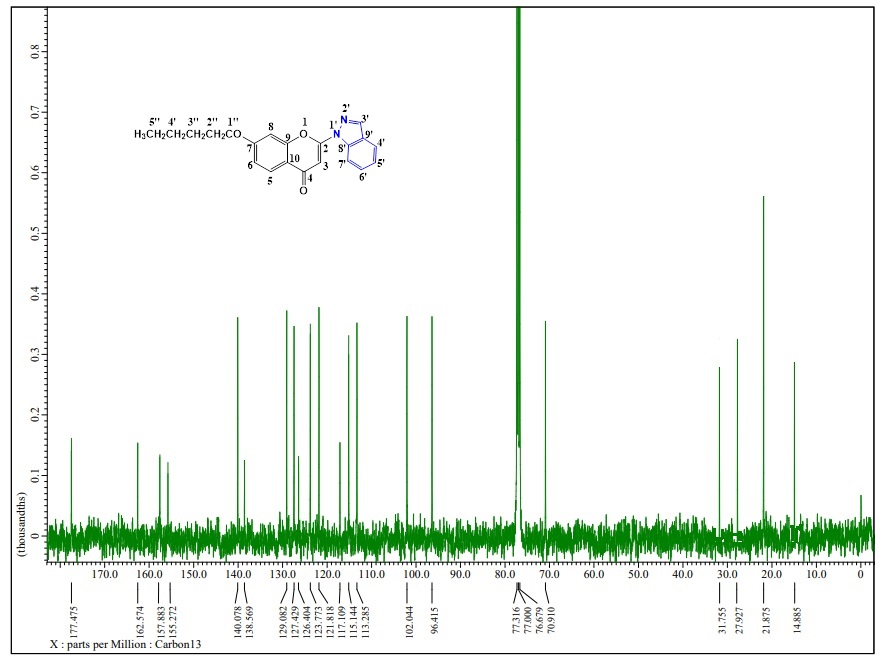


**Fig 14: ^13^C NMR of compound 6g**


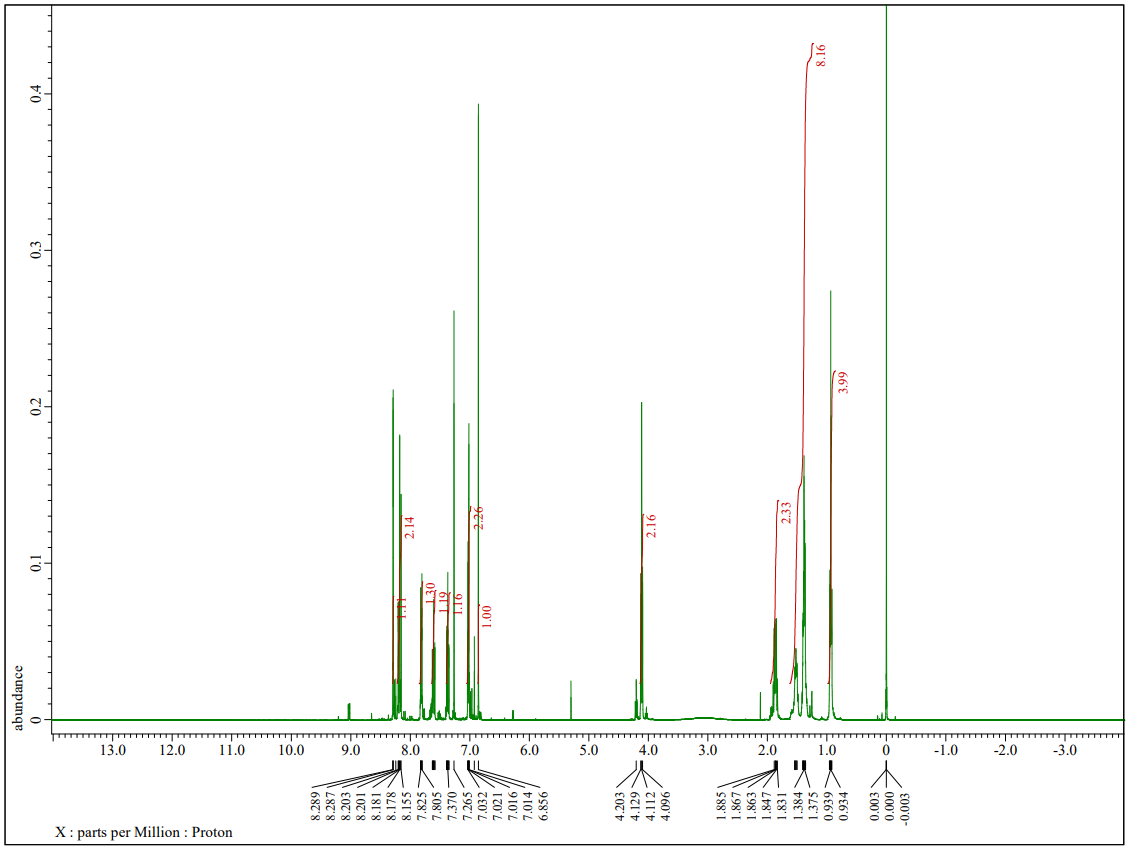


**Fig 15: ^1^H NMR of compound 6h**


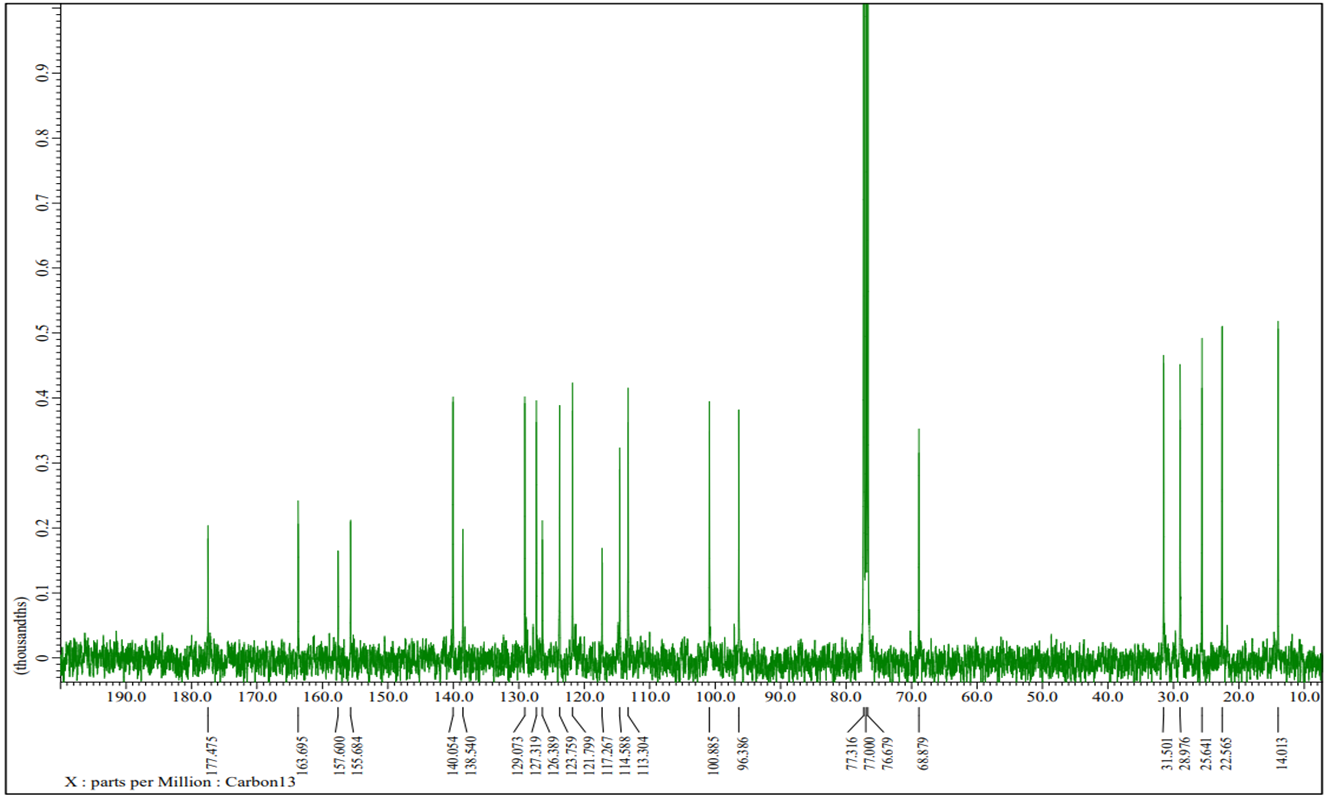


**Fig 16: ^13^C NMR of compound 6h**


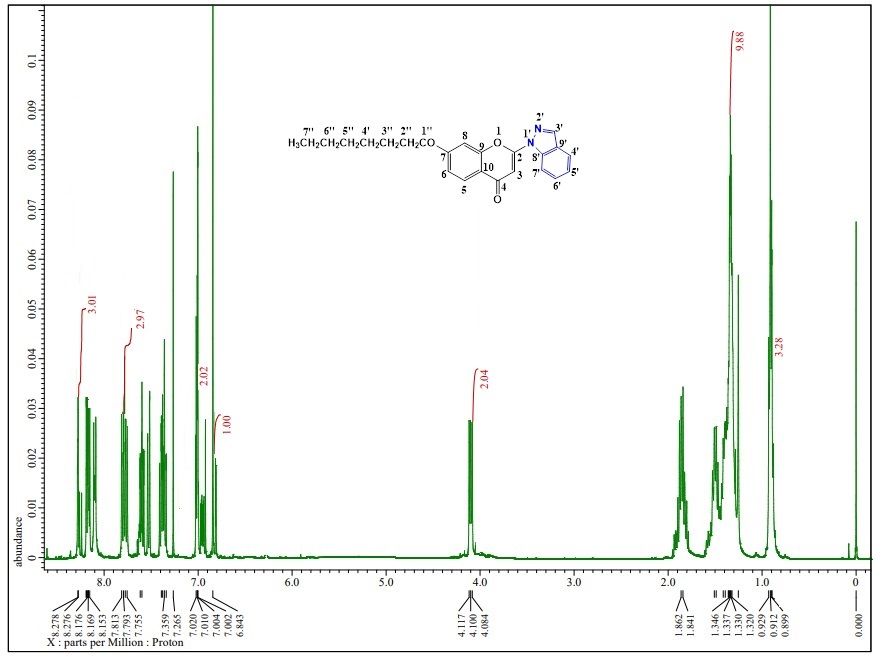


**Fig 17: ^1^H NMR of compound 6i**


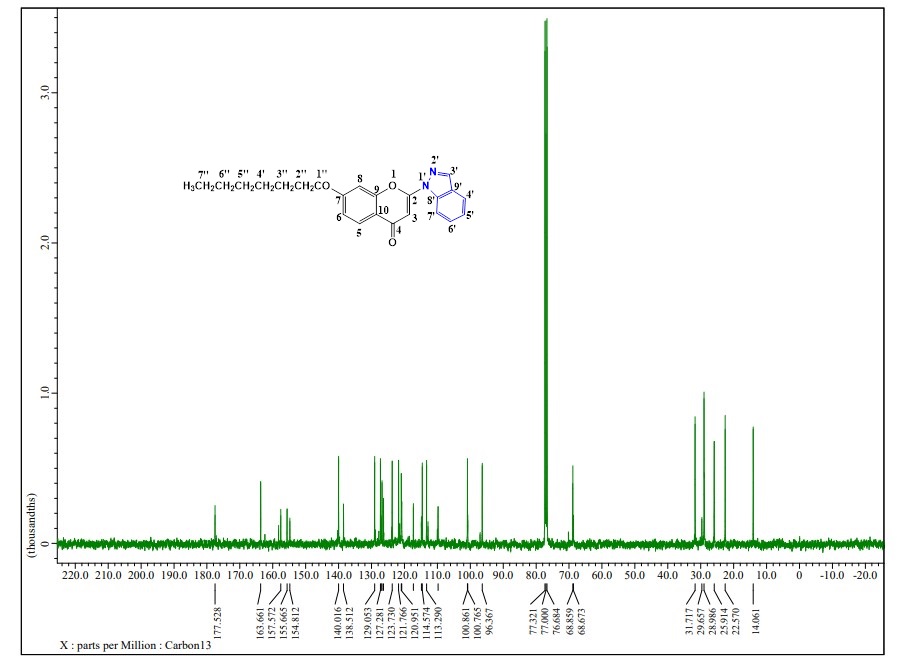


**Fig 18: ^13^C NMR of compound 6i**


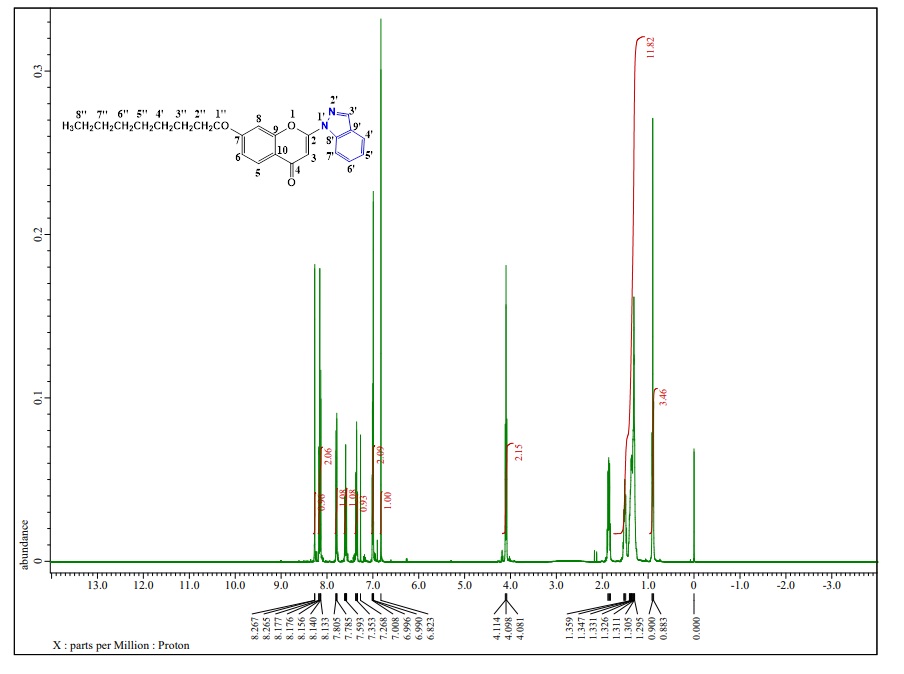


**Fig 19: ^1^H NMR of compound 6j**


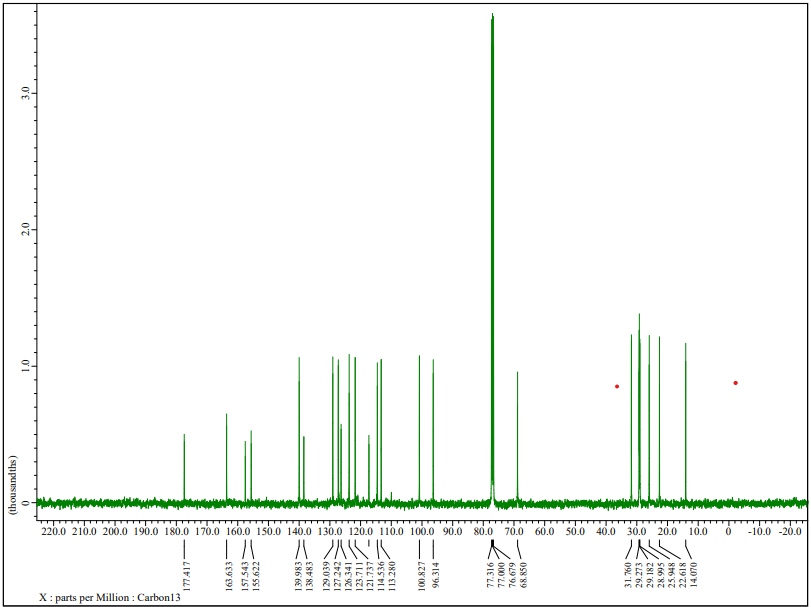


**Fig 20: ^13^C NMR of compound 6j**


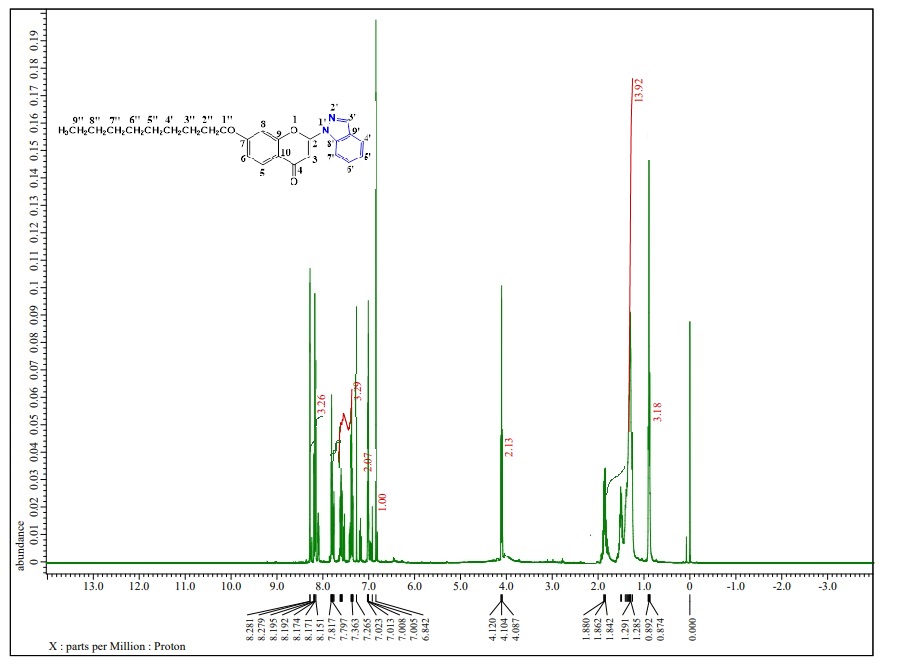


**Fig 21: ^1^H NMR of compound 6k**


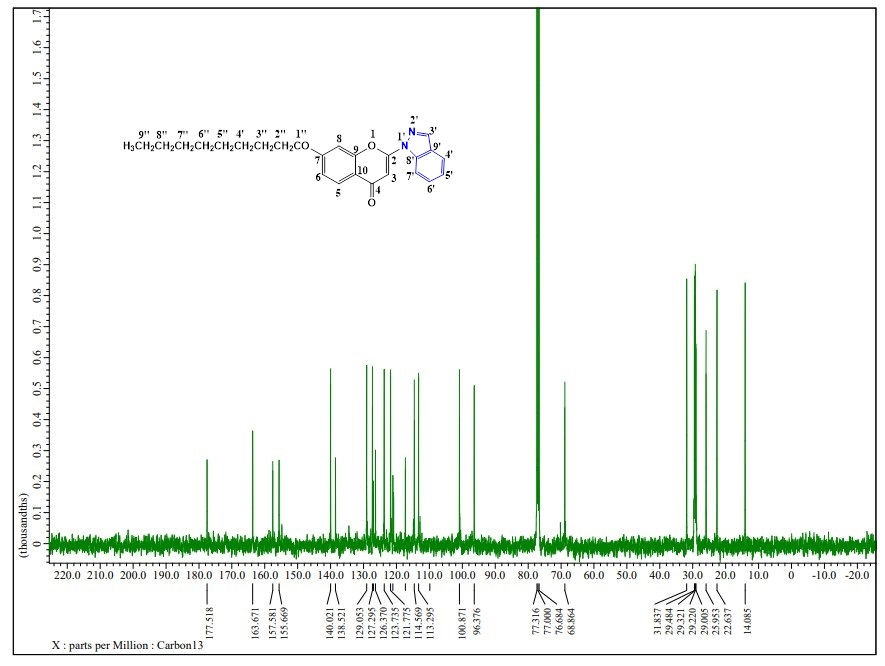


**Fig 22: ^13^C NMR of compound 6k**


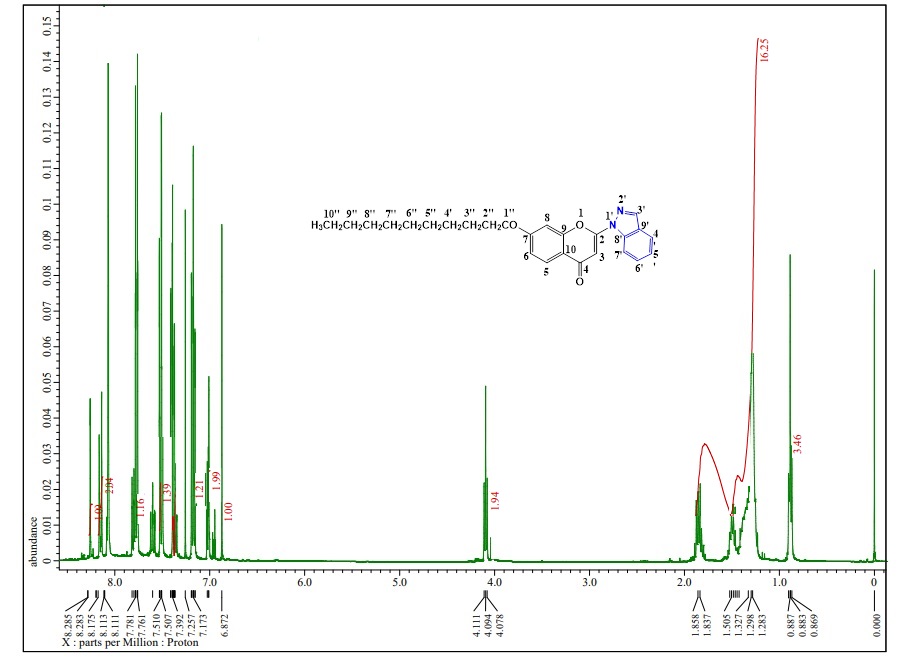


**Fig 23: ^1^H NMR of compound 6l**


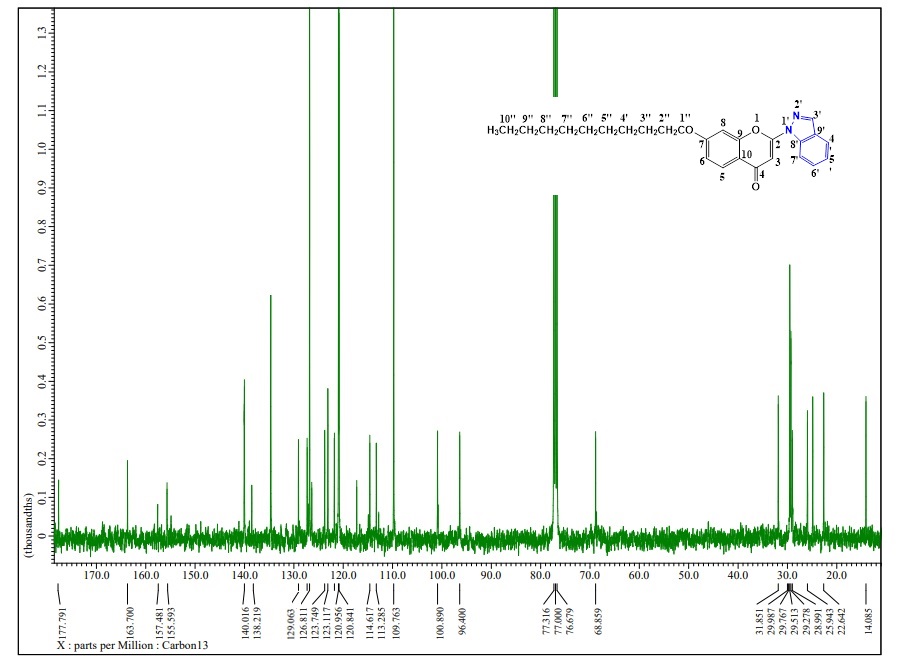


**Fig 24: ^13^C NMR of compound 6l**


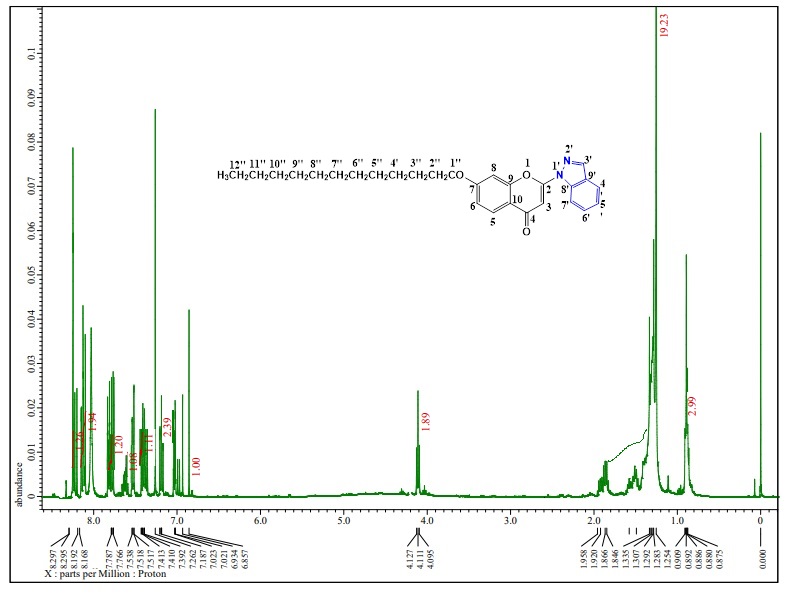


**Fig 25: ^1^H NMR of compound 6m**


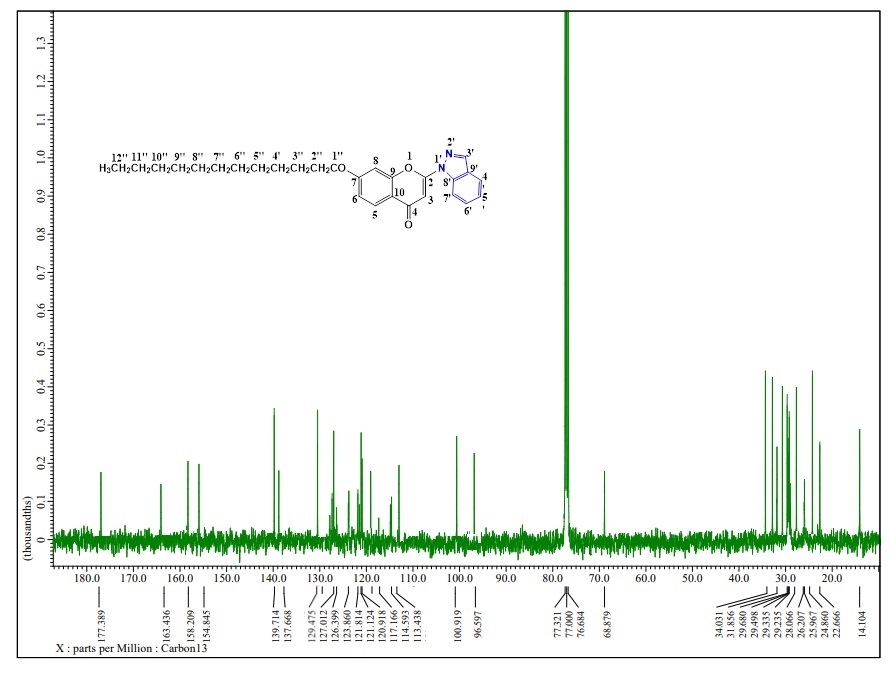


**Fig 26: ^13^C NMR of compound 6m**


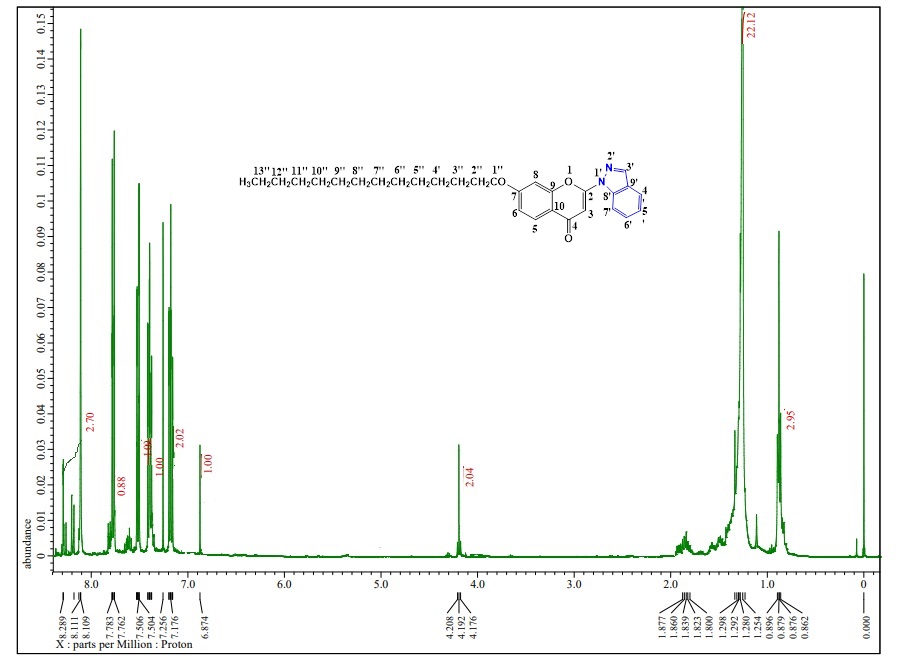


**Fig 27: ^1^H NMR of compound 6n**


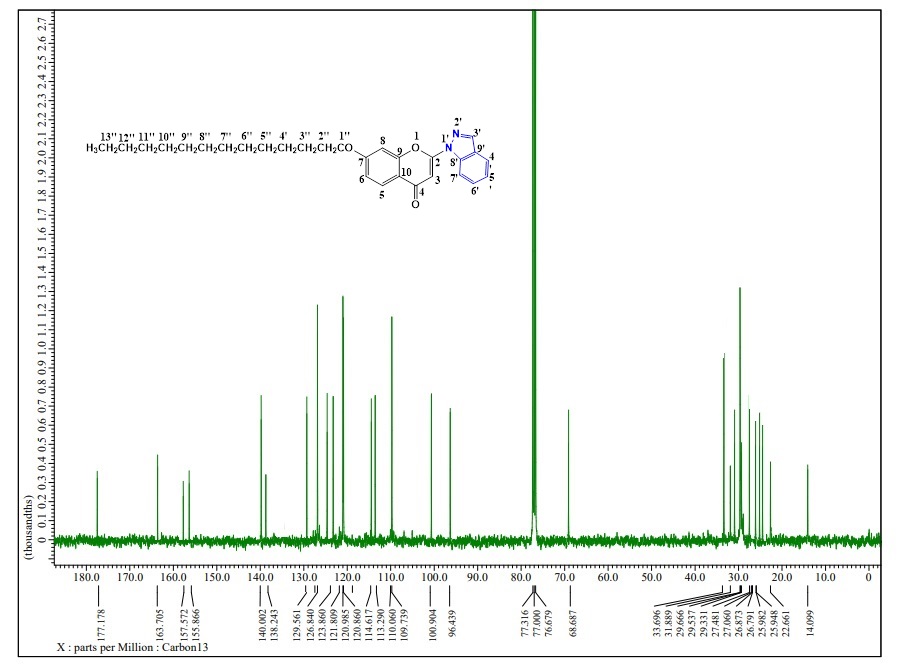


**Fig 28: ^13^C NMR of compound 6n**


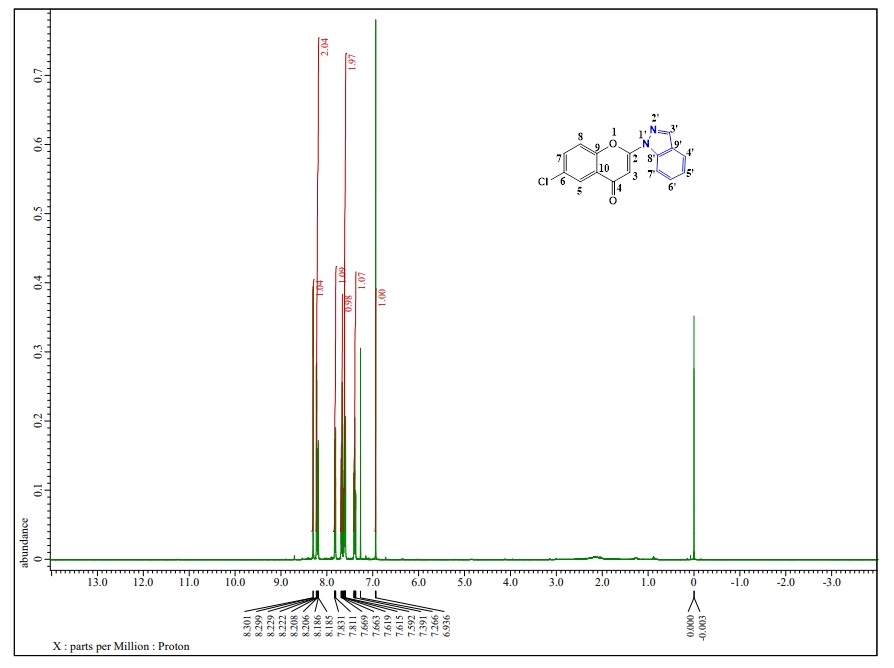


**Fig 29: ^1^H NMR of compound 6o**


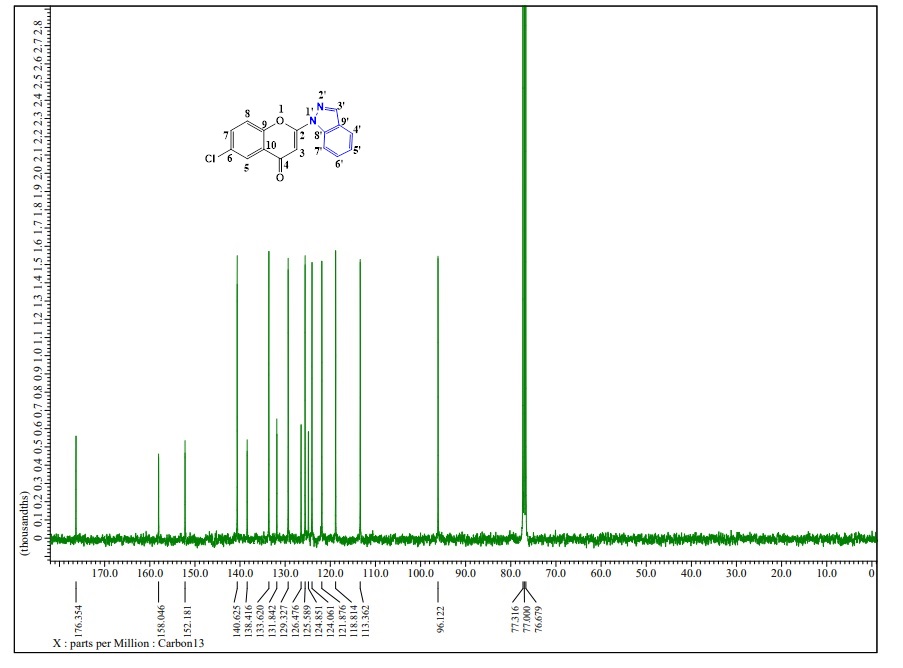


**Fig 30: ^13^C NMR of compound 6o**


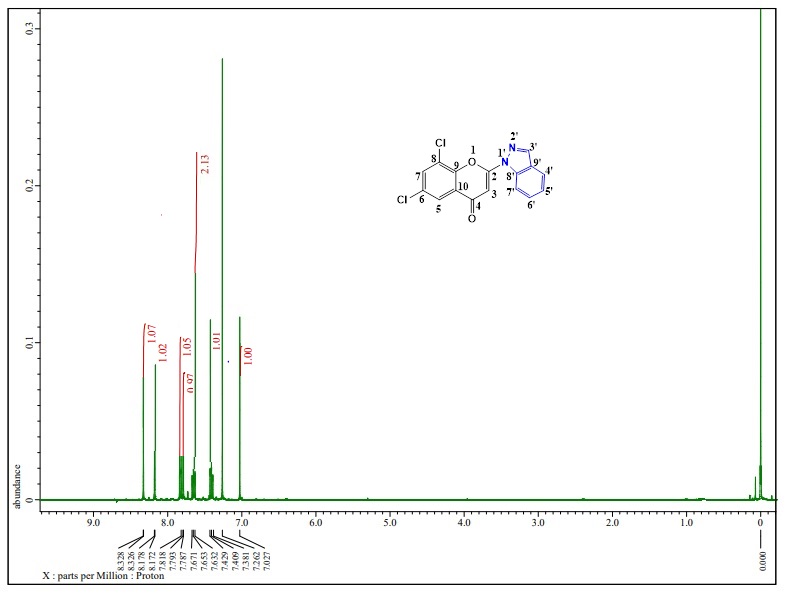


**Fig 31: ^1^H NMR of compound 6p**


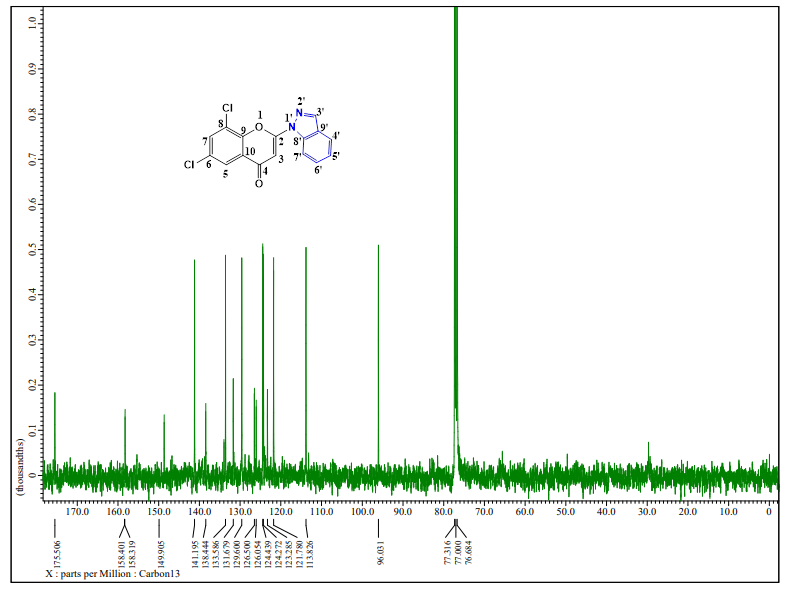


**Fig 32: ^13^C NMR of compound 6p**


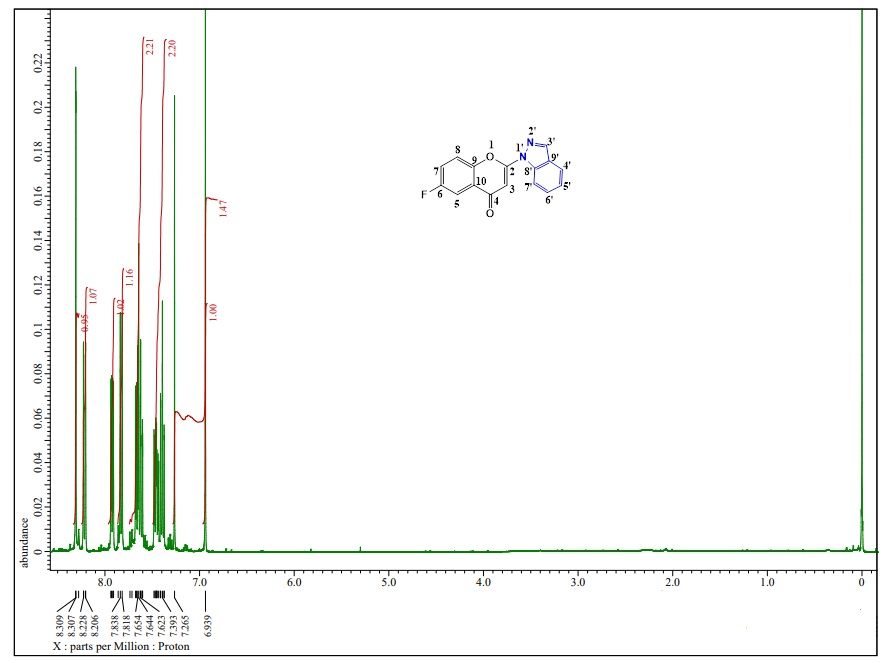


**Fig 33: ^1^H NMR of compound 6q**


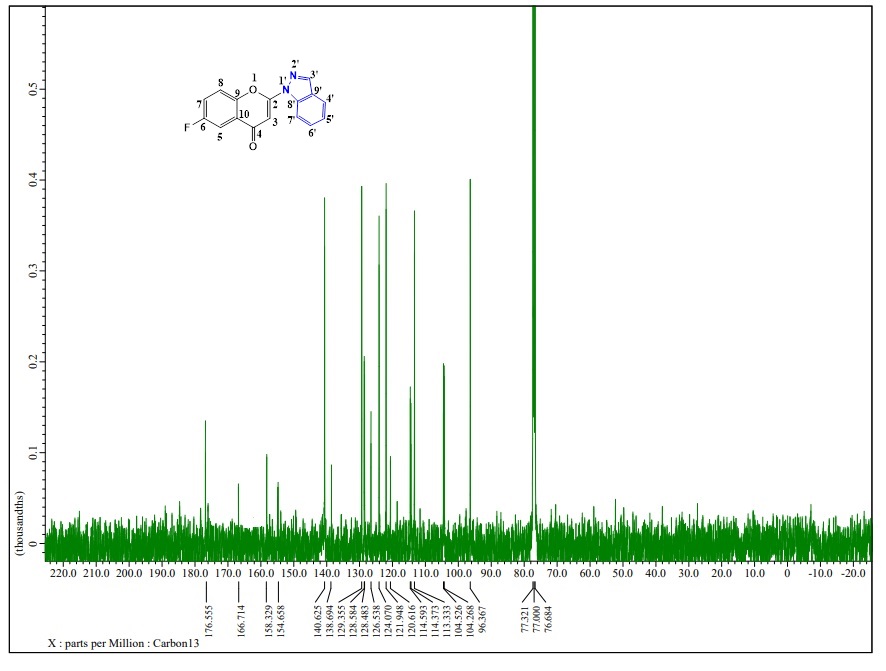


**Fig 34: ^13^C NMR of compound 6q**


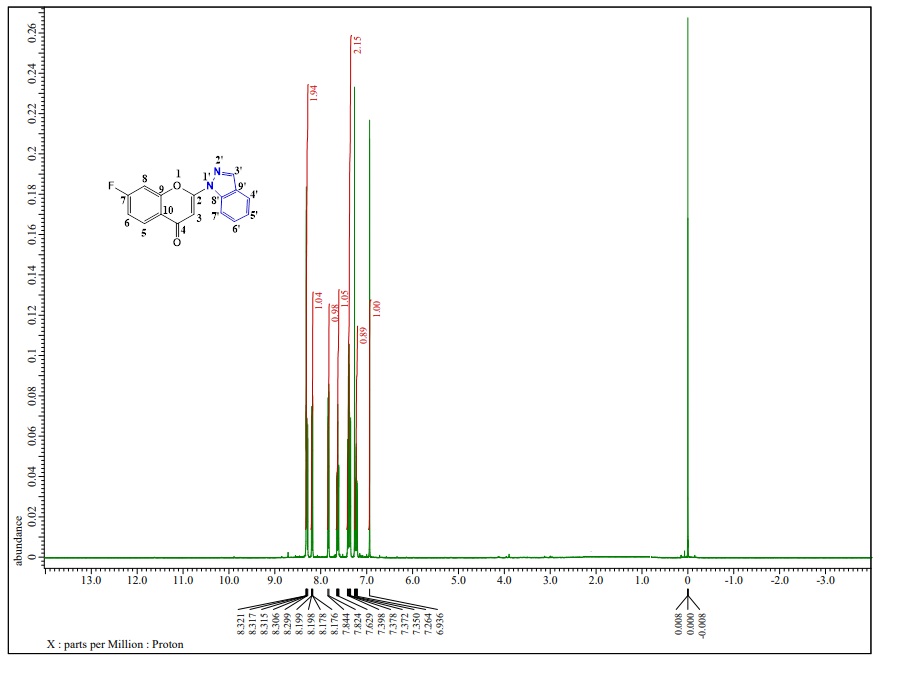


**Fig 35: ^1^H NMR of compound 6r**


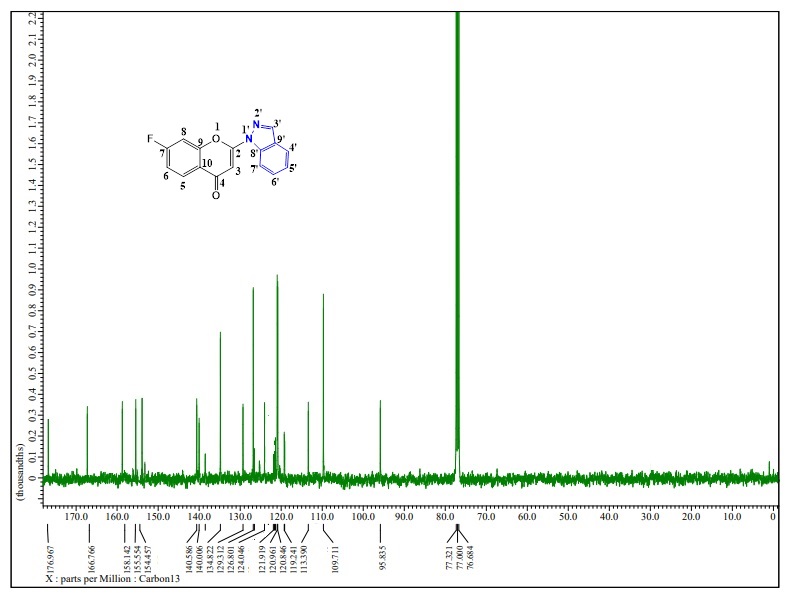


**Fig 36: ^13^C NMR of compound 6r**


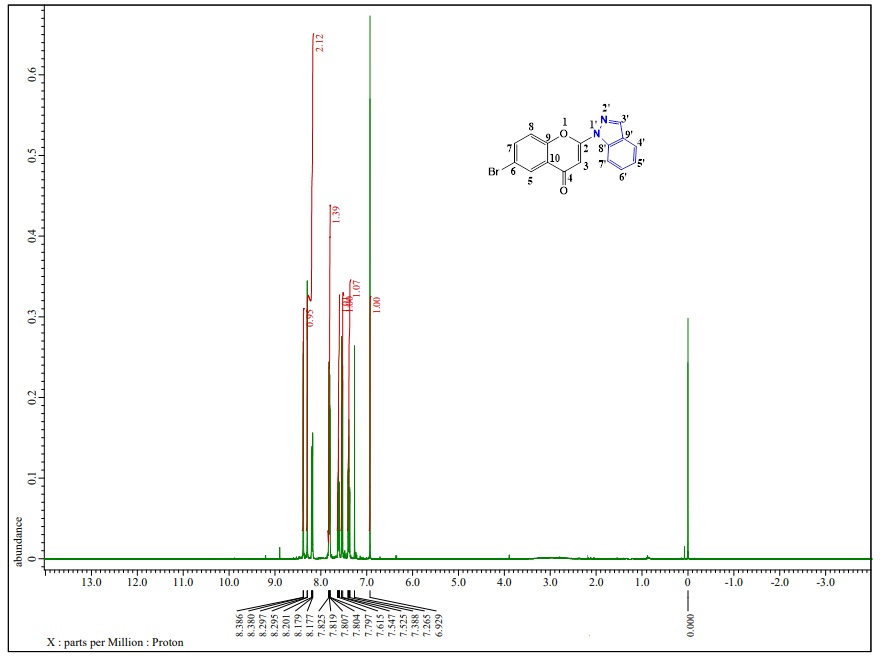


**Fig 37: ^1^H NMR of compound 6s**


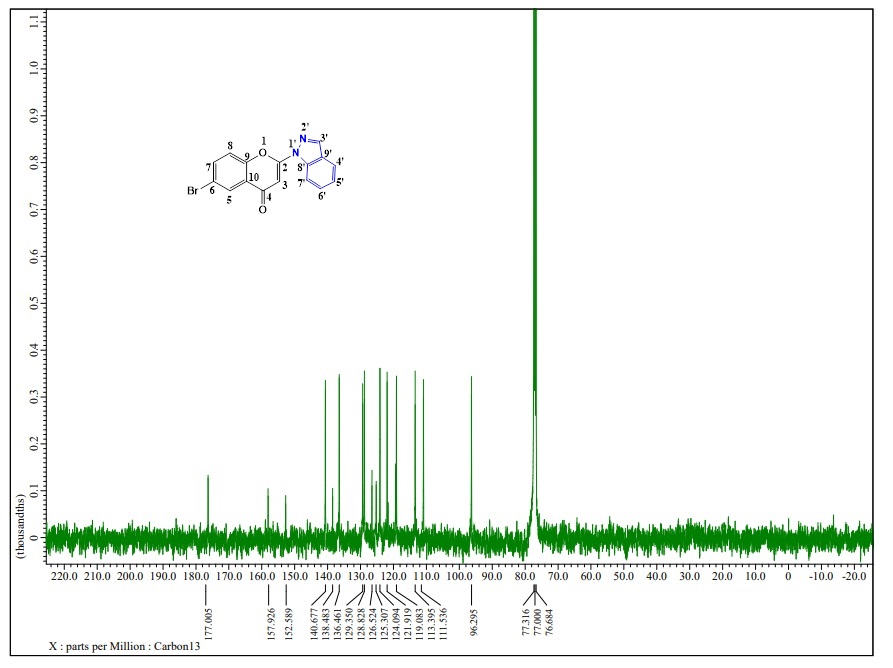


**Fig 38: ^13^C NMR of compound 6s**


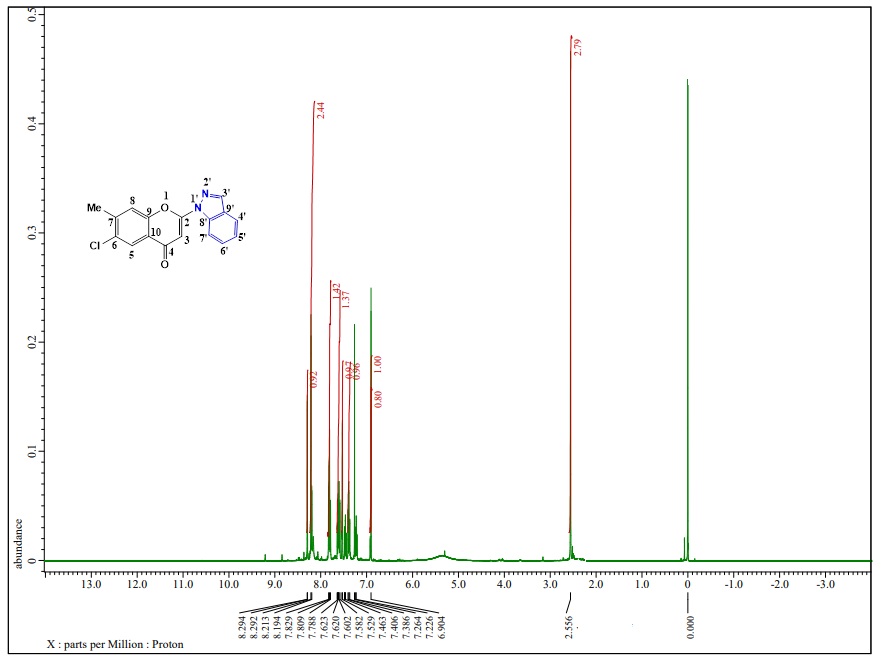


**Fig 39: ^1^H NMR of compound 6t**


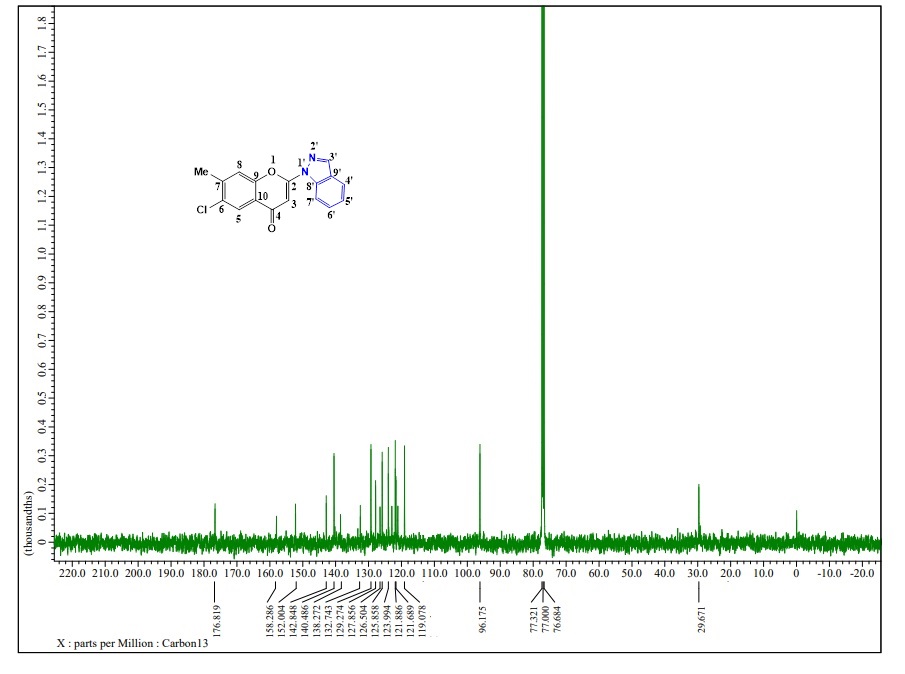


**Fig 40: ^13^C NMR of compound 6t**

**
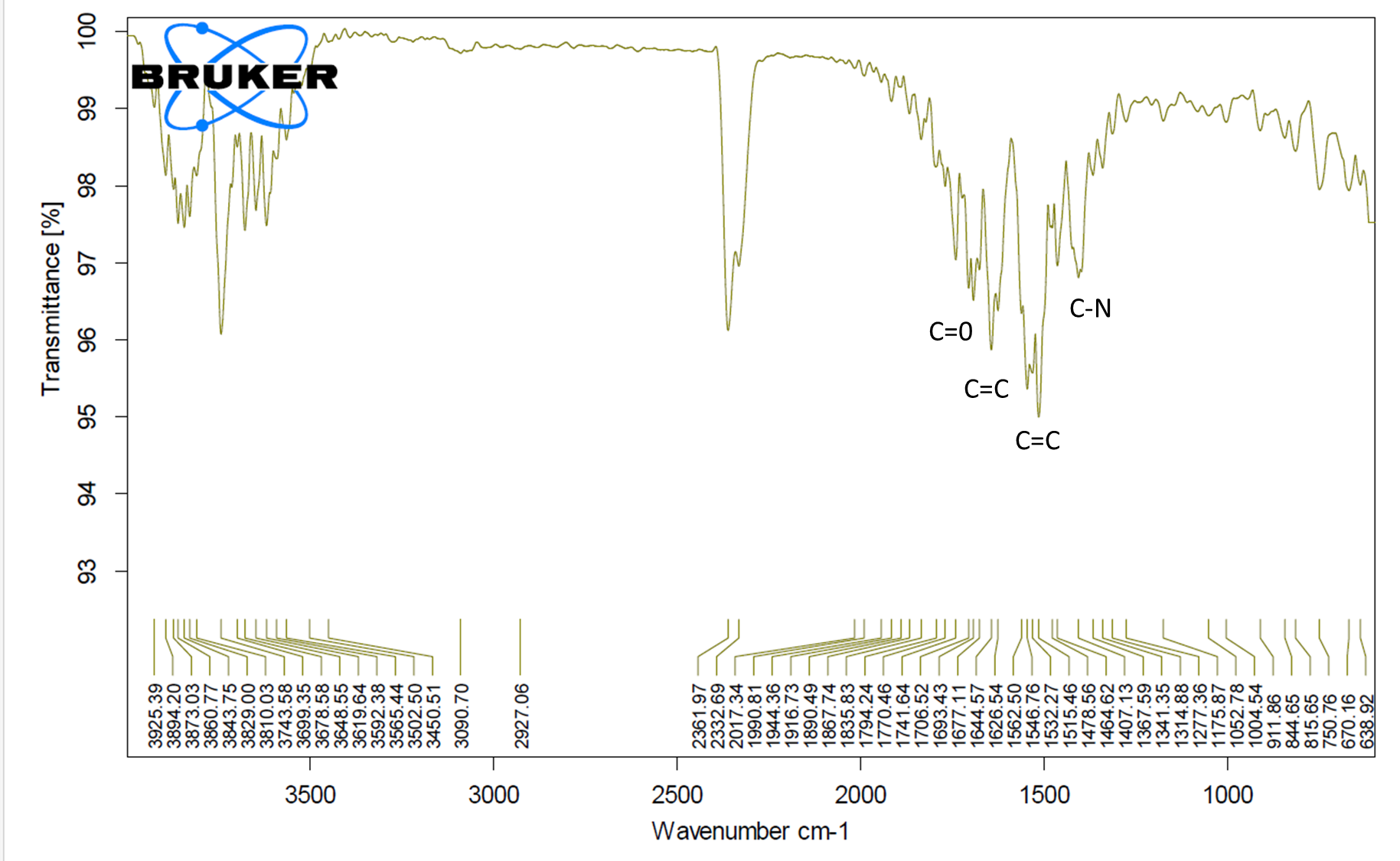
**

**Fig 41: Representative IR of indazolylchromones**
